# Shared Subgenome Dominance Following Polyploidization Explains Grass Genome Evolutionary Plasticity from a Seven Protochromosome Ancestor with 16K Protogenes

**DOI:** 10.1093/gbe/evt200

**Published:** 2013-12-06

**Authors:** Florent Murat, Rongzhi Zhang, Sébastien Guizard, Raphael Flores, Alix Armero, Caroline Pont, Delphine Steinbach, Hadi Quesneville, Richard Cooke, Jerome Salse

**Affiliations:** ^1^INRA/UBP UMR 1095 GDEC (Génétique, Diversité et Ecophysiologie des Céréales), Clermont Ferrand, France; ^2^INRA ‘Unité de Recherche en Génomique et Informatique’ Bat 18, Centre INRA de Versailles, Versailles, France; ^3^UMR5096 CNRS (Laboratoire Génome et Développement des Plantes), Université de Perpignan via Domitia, Perpignan, France

**Keywords:** synteny, evolution, genome, dominance, duplication, ancestor

## Abstract

Modern plant genomes are diploidized paleopolyploids. We revisited grass genome paleohistory in response to the diploidization process through a detailed investigation of the evolutionary fate of duplicated blocks. Ancestrally duplicated genes can be conserved, deleted, and shuffled, defining dominant (bias toward duplicate retention) and sensitive (bias toward duplicate erosion) chromosomal fragments. We propose a new grass genome paleohistory deriving from an ancestral karyotype structured in seven protochromosomes containing 16,464 protogenes and following evolutionary rules where 1) ancestral shared polyploidizations shaped conserved dominant (D) and sensitive (S) subgenomes, 2) subgenome dominance is revealed by both gene deletion and shuffling from the S blocks, 3) duplicate deletion/movement may have been mediated by single-/double-stranded illegitimate recombination mechanisms, 4) modern genomes arose through centromeric fusion of protochromosomes, leading to functional monocentric neochromosomes, 5) the fusion of two dominant blocks leads to supradominant neochromosomes (D + D = D) with higher ancestral gene retention compared with D + S = D (i.e., fusion of blocks with opposite sensitivity) or even S + S = S (i.e., fusion of two sensitive ancestral blocks). A new user-friendly online tool named “PlantSyntenyViewer,” available at http://urgi.versailles.inra.fr/synteny-cereal, presents the refined comparative genomics data.

## Introduction

**Fig. 1.—:**
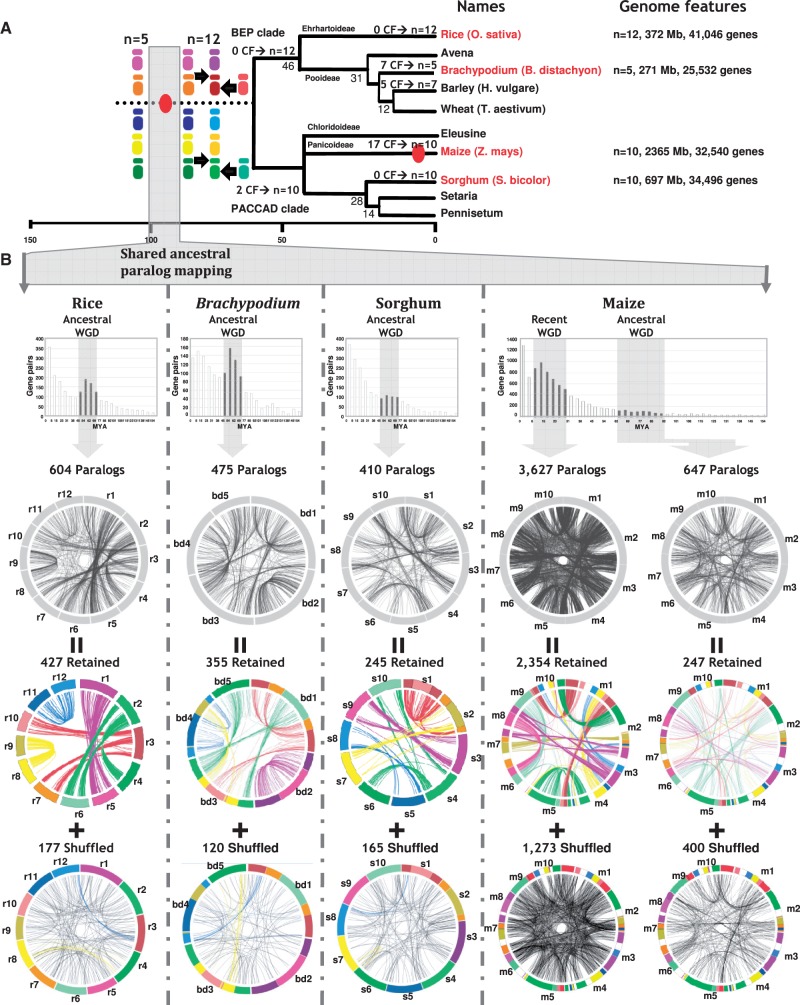
Retained and shuffled duplicated genes. (*A*) Grass genome evolutionary scenario. Divergence times from a common ancestor are indicated on the branches of the phylogenetic tree (in million years). WGD events are illustrated with red circles on the tree branches. The evolution of chromosome numbers of modern species from the ancestral genome structure is indicated, with the number of CF events. Genome features for the six cereal genomes investigated are mentioned at the right side of the figure with the number of chromosomes, physical size, and number of annotated unigenes. Ancestral karyotypes are differentiated by a color code that represents the *n* = 5 extinct ancestor and *n* = 12 ancestral intermediate (left). (*B*) Representation of retained and shuffled duplicates. Pairs of ancestral duplicated genes were identified based on *Ks* distribution (black bars). The duplicated genes corresponding to the ancestral tetraploidization (referenced as ancestral WGD) and neopolyploidization (referenced as recent WGD in the case of maize) have been mapped onto the four genomes (rice, *Brachypodium*, sorghum, and maize). Total numbers of duplicated genes (top circles with black connecting lines) are composed of duplicates retained on ancestral paralogous chromosomes (middle circle with colored connecting lines) and shuffled duplicates (bottom circles). Colored circular chromosomes and connecting lines illustrate their ancestral origin from five protochromosomes, whereas nonsyntenic duplicated genes are linked with black lines.

Genome sequences from flowering plants that are derived from a common ancestor 135–250 Ma are increasingly available for evolutionary studies. Recent access to monocot genome sequences from both the Bambusoideae–Ehrhartoidea–Pooideae (BEP) and Panicoideae–Aristidoideae–Centhothecoideae–Chloridoideae–Arundinoideae–Danthoideae (PACCAD) clades allowed paleogenomic analyses aimed at reconstructing genome paleohistory from ancestors. Comparative analysis of these monocot sequences, including Panicoideae (sorghum [Paterson et al. 2009]maize [Schnable et al. 2009]), Ehrhartoideae (rice [International Rice Genome Sequencing Project 2005]), and Pooideae (*Brachypodium* [International *Brachypodium* Initiative 2010]), suggested that grasses derive from *n* = 5 to 12 ancestral karyotypes (named AGK for ancestral grass karyotypes) containing 6,045 ordered protogenes with a minimum physical size (i.e., cumulative coding gene space) of 33 Mb (Salse et al. 2008; Salse, Abrouk, Bolot, et al. 2009; Salse, Abrouk, Murat, et al. 2009 Salse et al. 2012; Bolot et al. 2009), cf. figure 1*A*. Modern grass genomes were then shaped from this AGK through whole genome duplication (WGD) followed by ancestral chromosome fusion (CF); for review, Salse 2012). It is now well established that almost all modern diploid grass species are paleopolyploids, following at least two shared ancestral duplication events (Paterson et al. 2004; Tang et al. 2008; Van de Peer et al. 2009; Tang et al. 2010; Jiao et al. 2011; Wang et al. 2011).

Polyploidization has been shown to be followed by genome-wide diploidization (also referenced as partitioning) through differential elimination of duplicated gene redundancy at the whole genome and gene levels (Wang et al. 2005; Schnable, Freeling, et al. 2012; Schnable, Wang, et al. 2012). Gene-based diploidization acts at the structural (duplicated gene deletion) and functional (duplicated gene neo- or subfunctionalization) levels (for review, Salse 2012 and Freeling et al. 2012). At the structural level, it has been shown that protein-coding genes behave differently in response to this diploidization process. Diploidization-resistant genes, mainly transcription factors (TFs) or regulators (TRs), are retained as duplicates following WGDs, whereas other genes are considered diploidization sensitive and return to a singleton status via selective gene deletion (Thomas et al. 2006; Sankoff et al. 2010; Pont et al. 2011; Abrouk et al. 2012). The loss of diploidization sensitive genes is not random at the whole genome level, leading to dominant (reduced duplicated gene loss) and sensitive (enhanced duplicated gene loss) subgenomes in paleo- or neopolyploids (Chang et al. 2010; Woodhouse, Schnable, et al. 2010; Schnable, Freeling, et al. 2012; Schnable, Wang, et al. 2012). At the functional level, recurrent gene or genome duplications generate functional redundancy followed by pseudogenization (unexpressed or functionless paralogs), concerted evolution (maintained function of paralogs), subfunctionalization (partitioned function of paralogs), or neofunctionalization (novel function of paralogs) of the retained diploidization resistant genes during grass genome evolution (Moore and Purugganan 2005; Edger and Pires 2009; Wang et al. 2012). This divergence, either by subfunctionalization or neofunctionalization, has been proposed as one of the most important sources of evolutionary novelty in living organisms (Doyle et al. 2008), with total functional diploidization observed after 50 Myr following WGDs (Pont et al. 2011). WGD thus constitutes a recurrent source of redundant genes that can be lost (structural partitioning) or co-opted for novel functions/expression patterns (functional partitioning), increasing the potential for niche specialization or morphological innovation (De Bodt et al. 2005).

Up to now, diploidization following WGD has been investigated in plants either at the whole genome level in single species (such as maize and rice [Schnable, Freeling, et al. 2012] for the monocots, Brassicaceae and Arabidopsis [Schnable, Wang, et al. 2012] for the dicots) or at the gene family level for several species (such as microRNA in grasses [Abrouk et al. 2012]). An exhaustive multispecies investigation of the diploidization mechanism at the whole genome and gene levels to gain precise insights into structural and functional evolution of duplicated genes and chromosomes is still lacking. Comparing maize, rice, *Brachypodium* and sorghum genomes, we have investigated the role of diploidization in reducing structural and functional duplicated gene redundancy in paralogous blocks. Although biased deletion of duplicated genes is reported in the literature as part of the diploidization process, defining dominant and sensitive chromosomal blocks (for review, Salse 2012 and Freeling et al. 2012), we describe here the differential evolution of dominant and sensitive blocks derived from ancient and recent polyploidies during grass paleohistory. Overall, taking into account retained, shuffled, and deleted duplicated genes in our paleogenomics investigation, we precisely define ancestral and modern dominant and sensitive chromosomal regions and propose a novel evolutionary scenario at the genome, chromosome, and gene levels from an ancestral karyotype structured in seven protochromosomes containing 16,464 protogenes, following newly defined evolutionary rules.

## Materials and Methods

### Identification of Conserved versus Shuffled Ancestral Duplicated Genes

Plant genomes (41,046 genes in rice, 25,532 genes in *Brachypodium*, 34,496 genes in sorghum, and 32,540 genes in maize, cf. table 1*A*) were compared through annotated CoDing Sequences (CDS) alignments (using Blast). To increase the significance of interspecific sequence alignments for inferring evolutionary relationships between genomes, we used parameters previously defined from Blast results (Salse, Abrouk, Murat, et al. 2009). Briefly, grass CDS were compared using three parameters: Aligned length (AL = ∑ high scoring pairs [HSP] lengths), cumulative identity percentage (CIP = ∑ nb ID by [HSP/AL] × 100), and cumulative alignment length percentage (CALP = AL/query length). The CIP is the cumulative percentage of sequence identity observed for all the HSPs divided by the cumulative AL, that is, the sum of all HSP lengths. CALP is the sum of all HSP lengths (AL) divided by the length of the query sequence. These two thresholds were used to compare grass genomes depending on their evolutionary relationships: CIP/CALP of 70% and 50% for genomes deriving from common ancestors dating back to <50 Ma (i.e., closely related) and >50 Ma (i.e., distantly related), respectively (Salse, Abrouk, Murat, et al. 2009). Conserved and shuffled duplicates are defined as gene pairs having the same dating value (see next section) for which both or one copy, respectively, are found in known orthologous or paralogous blocks (Salse, Abrouk, Murat, et al. 2009), as shown in table 1 (i.e., lines and column corresponding respectively to orthologous and paralogous relationships between grass chromosomes).

**Table 1:** Duplicated Genes Derived from Paleo- and Neopolyploidization Events in Grasses

**Ancestors**			**Ancient Shared Paleoduplication**											
**Ancest. *n* = 5**	**Ancest. *n* = 7**	**Ancest. *n* = 12**	**Maize**			**Rice**			***Brachypodium***			**Sorghum**		
			**Chr**	**Orthol. Number**	**Paral. Number**	**Chr**	**Orthol. Number**	**Paral. Number**	**Chr**	**Orthol. Number**	**Paral. Number**	**Chr**	**Orthol. Number**	**Paral. Number**
*A*. Characterization of Ancestral Duplication in Maize, Rice, Sorghum, and Brachypodium														
**A5**	**A5**	**A5 (S)**	**6-8**	**309**	**61**	**5**	**496**	**129**	**2**	**412**	**106**	**3**	**353**	**59**
		**A1 (D)**	**3-8**	**740**		**1**	**1107**		**2**	**959**		**9**	**875**	
**A4**	**A4**	**A4 (D)**	**2-10**	**163**	**90**	**4**	**571**	**137**	**5**	**507**	**110**	**6**	**447**	**69**
		**A2 (D+S)**	**4-5**	**549**		**2**	**776**		**3**	**670**		**4**	**643**	
	**A6**	**A6 (S)**	**5-5-9**	**260**		**6**	**366**		**1**	**325**		**10**	**286**	
**A7**	**A7**	**A7 (S)**	**2-7**	**301**	**59**	**7**	**441**	**86**	**1**	**375**	**74**	**2**	**272**	**70**
		**A3 (D+D)**	**1-9-5**	**612**		**3**	**870**		**1**	**757**		**1**	**672**	
	**A10**	**A10 (S)**	**1-9-5**	**175**		**10**	**323**		**3**	**258**		**1**	**214**	
**A8**	**A8**	**A8 (S)**	**1-4-6-10**	**211**	**31**	**8**	**357**	**47**	**3**	**316**	**33**	**7**	**266**	**24**
		**A9 (D)**	**2-7**	**341**		**9**	**497**		**4**	**453**		**2**	**396**	
**A11**	**A11**	**A11 (D)**	**2-4**	**76**	**6**	**11**	**168**	**28**	**4**	**132**	**32**	**5**	**105**	**23**
		**A12 (S)**	**1-3-10**	**57**		**12**	**130**		**4**	**106**		**8**	**83**	
**Total gene number in blocks**				**3,794**	**↑**		**6,102**	**↑**		**5,270**	**↑**		**4,612**	**↑**
**Total retained duplicates (% /total dup.)**					**247 (38%)**			**427 (71%)**			**355 (75%)**			**245 (60%)**
**Total shuffled duplicates (% /total dup.)**					**400 (62%)**			**177 (29%)**			**120 (25%)**			**165 (40%)**
**Total duplicatedgenes (‰ /gene content)**					**647 (45‰)**			**604 (32‰)**			**475 (37‰)**			**410 (24‰)**

Ancestors			Recent Maize-Specific Neoduplication										
Ancestor *n* = 5	Ancestor *n* = 7	Ancestor *n* = 12	Maize Duplicate 1 (Dominant)			Maize Duplicate 2 (Sensitive)								
			Chr	Gene Number	Ortholog Number	Chr	Gene Number	Ortholog Number	Paralog Number					
*B*. Characterization of Recent Species-Specific Duplication in Maize														
**A5**	**A5**	**A5 (S)**	**6**	**1,262**	**203**	**8**	**995**	**293**	**175**					
		**A1 (D)**	**3**	**2,743**	**479**	**8**	**1,716**	**124**	**379**					
**A4**	**A4**	**A4 (D)**	**2**	**1,810**	**274**	**10**	**1,023**	**171**	**219**					
		**A2 (D+S)**	**5**	**2,313**	**419**	**4**	**1,366**	**209**	**283**					
	**A6**	**A6 (S)**	**9_5**	**1,647**	**253**	**6**	**869**	**113**	**151**					
**A7**	**A7**	**A7 (S)**	**7**	**1,470**	**259**	**2**	**701**	**113**	**155**					
		**A3 (D+D)**	**1**	**2,830**	**578**	**9_5**	**1,627**	**308**	**494**					
	**A10**	**A10 (S)**	**1**	**664**	**119**	**9_5**	**417**	**65**	**85**					
**A8**	**A8**	**A8 (S)**	**1-6-10**	**1,172**	**168**	**4**	**823**	**110**	**108**					
		**A9 (D)**	**7**	**817**	**151**	**2**	**652**	**90**	**110**					
**A11**	**A11**	**A11 (D)**	**4**	**596**	**75**	**2**	**296**	**28**	**23**					
		**A12 (S)**	**1-10**	**813**	**49**	**3**	**387**	**58**	**63**					
**Total gene number in blocks**				**18,137**	**3,027**		**10,872**	**1,682**	**↑**					
**Retained neoduplicated genes (% /total dup.)**									**2,354 (62%)**					
**Shuffled neoduplicated genes (% /total dup.)**									**1,273 (38%)**					
**Total neoduplicated genes (‰ /gene content)**									**3,627 (121‰)**					

Paral. = Paralogs, Orthol. = Orthologs.

### Ancestral Karyotype and Protogene Order Reconstruction

Ancestral karyotypes (protochromosomes) were reconstructed by computing common intervals of conserved blocks between two genomes, based on validated orthologous genes/blocks, or within a single genome, using validated paralogous genes/blocks, to obtain contiguous ancestral regions (CARs) (Salse 2012). Orthologous genes defined by CDS alignment were grouped into synteny groups using DRIMM-Synteny or Cynteny (both tools providing similar results) with the following “cleaning” parameters: minimum number of genes in synteny blocks (>5), maximum gap between two synteny blocks (100 kb), and minimum length of synteny blocks (100–500 kb). The derived postduplication ancestor (*n* = 12) was used to identify the preduplication (*n* = 5–7) ancestral karyotypes. Chromosomal blocks that are duplicated in two different genomes but located at orthologous positions when comparing the two genomes are considered to be unique in the ancestor (i.e., CAR) and derived from a shared prespeciation duplication event. In contrast, a chromosomal block that is duplicated in one genome but not identified as duplicated at orthologous positions when comparing two genomes is considered to be species specific, resulting from a postspeciation duplication event. The same approach was applied for all types of identified rearrangements, including inversions and translocations characterized as ancestral or lineage specific. From the identified CARs, the most likely evolutionary scenario is proposed on the following assumptions: 1) ancestor modeling is based on duplications (or any shuffling events) found at orthologous positions between modern species and thus considered as ancestral, 2) evolutionary history is based on the smallest number of shuffling operations (including inversions, deletions, fusions, fissions, translocations) that explain evolution from the reconstructed ancestral genome to modern karyotypes. Reconstruction of the ancestral gene order within protochromosomes of the ancestral karyotypes can be performed using several public methods, such as InferCARs (Ma et al. 2006), MGRA (Multiple Genome Rearrangements and Ancestors, Alekseyev and Pevzner 2009) or ANGES (ANcestral GEnomeS, Jones et al. 2012). ANGES is similar to InferCARs in principle, but is more general as it computes both ancestral adjacencies and intervals (only adjacencies for InferCARs), and has been tested on a wide range of kingdoms: plants, animals, bacteria, fungi (in contrast to InferCARs, only tested on mammals). Among the available tools, only ANGES (Jones et al. 2012) allows gene loss and was used in the current study to reconstruct the ancestral gene order in the grass ancestors. It was used to order synteny groups (obtained by DRIMM-Synteny or Cynteny) with each other in CARs, producing an ancestral gene order for AGK (*n* = 12). The ancestral gene order for the pre-duplication ancestor (*n* = 7) consists in only remaining duplicated genes characterized in the post-duplication ancestor (*n* = 12).

### Dating of Duplication and Speciation Events

We dated sequence divergence and speciation and duplication events using the rate of synonymous (*Ks*) substitutions. The average substitution rate (*r*) of 6.5 × 10^−^^9^ substitutions per synonymous site per year is classically used to calibrate the ages of the considered paralogous and orthologous genes. The time (*T*) since gene insertion is then estimated using the formula *T* = *Ks*/2*r*. *Ks* between paralogs was modeled as mixtures of log-transformed exponentials and normals, representing recent and ancient WGDs. *Ks* distribution can be then described as mixtures of log-normal components that represent single (for rice, *Brachypodium,* and sorghum) or multiple (for maize) rounds of genome duplications, using the EMMIX software (http://www.maths.uq.edu.au/∼gjm/emmix/emmix.html, last accessed December 24, 2013). The EMMIX mixed populations were modeled with one component (referenced as centroids) for rice, *Brachypodium* and sorghum and two components for maize. We finally selected one best mixture model for each round of duplication on the basis of the Bayesian information criterion, with an additional restriction on the mean/variance structure for *Ks* (Cui et al. 2006).

### Characterization of Dominant (D) and Sensitive (S) Fragments

The known duplicated regions conserved in grasses were compared for their retention of ancestral genes. For each pair of ancestral duplicated chromosomes, we characterized the number of retained ancestral genes (i.e., genes that are conserved between the investigated grass species, cf. table 1) and defined dominant (highest number of retained genes) and sensitive (lowest number of retained genes) chromosomal blocks. To validate the observed partitioning and the variance of gene retention/deletion without subgenome dominance (Ho: duplicated gene deletion is random between paralogous chromosomes), we compared observed values (i.e., numbers of retained genes on duplicated blocks) and expected or simulated values (i.e., equal distribution of the total number of observed retained genes between the two blocks) using a χ^2^ test. If the *P* value was lower than 0.05, we rejected the null hypothesis and considered that expected and observed values were significantly different, i.e., biased retention of duplicates or subgenome dominance is statistically validated.

### Gene Ontology Analysis

We used the AgriGO website (http://bioinfo.cau.edu.cn/agriGO/analysis.php, last accessed December 24, 2013) to identify gene ontologies (GOs) for our subsets of genes located on dominant and sensitive chromosomal blocks following both paleo- and neoduplications. The same site was also used to identify GOs that are over- or underrepresented in dominant and sensitive blocks of each species, taking into account the whole genome GO distribution.

## Results

### Characterization of Retained, Deleted, and Shuffled Duplicated Genes

We previously proposed an evolutionary model of the grass genomes, based on the identification of seven ancestral shared duplicated blocks in wheat, maize, *Brachypodium*, sorghum, and rice, in which grasses underwent a whole genome paleotetraploidization event 50–70 Ma (Salse, Abrouk, Bolot, et al. 2009; Salse, Abrouk, Murat, et al. 2009; Salse et al. 2012; Murat et al. 2010, 2012). In this scenario, grasses derived from an *n* = 5 ancestor that went through a WGD to reach an *n* = 12 (A1–A12 CARs) intermediate, figure 1*A*. Modern grass genomes were proposed to derive from this duplicated intermediate (i.e., a mosaic of A1-A5, A2-A4, A2-A6, A3-A7, A3-A10, A8-A9, A11-A12 paralogous ancestral blocks) through distinct ancestral CF patterns (cf. CF number on branches in fig. 1*A*). Here, to distinguish precisely gene pairs that have been retained at ancestral positions (still mapped on the known sister paralogous regions) and those that have been shuffled to nonorthologous sites, we aligned four genomes (rice, *Brachypodium*, sorghum, maize) against themselves to identify homologous genes (see Materials and Methods). We identified a total of 2,379, 1,608, 2,337, and 11,366 duplicated genes, respectively, in rice, *Brachypodium*, sorghum, and maize (fig. 1*B,* top, *Ks* distribution). We considered gene pairs dating between 50 and 70 Ma (shown as black bars in the *Ks* distribution on fig. 1*B*) to be ancient duplicates, identifying 604, 475, 410, and 647 duplicates in the rice, *Brachypodium*, sorghum, and maize genomes, respectively. Genomic positions of these pairs are shown in the top circles of figure 1*B*: 427, 393, 245, and 247 pairs are still located in ancestral conserved duplicated blocks (colored connecting lines in middle circles of fig. 1*B*, referenced as retained duplicates), respectively, in the rice, *Brachypodium*, sorghum, and maize genomes. However, 177 (29%), 120 (25%), 165 (40%), and 400 (62%) pairs from the same genomes are found at nonsyntenic locations (black connecting lines in the bottom circles of fig. 1*B* referenced as shuffled duplicates) although they derive from the same paleotetraploidization event based on the dating procedure. These genes have distinct functions and cannot be associated with recent large and fast-evolving duplicated gene families.

Ancestral duplicates that are no longer detectable at orthologous positions in grasses correspond to lineage-specific shuffling events that involve single genes or a few genes in clusters. Although a general pattern of single copy-based gene movement is observed, 12‰ (10 genes out of the 824 nonsyntenic ancestral duplicates) have moved by groups of two to four genes, and two much larger blocks were identified, involving 5,452 genes in total in rice, *Brachypodium,* and sorghum, including 83 duplicates. These two blocks were first described in rice but were not identified in sorghum (Paterson et al. 2004). However, although they do not correspond to the known reported ancestral duplicated protochromosomes (see the five-color code, fig. 1*A* left), we find them at orthologous positions in all grasses (r4–8 and r3–12 in rice, b3–5 and b1–4 in *Brachypodium*, s6–7 and s1–8 in sorghum, m1/4–2/10, and m1/9–3/10 in maize). They are highlighted in yellow (A4–A8) and blue (A3–A12) on the nonsyntenic duplicate circles (fig. 1*B*, bottom). 

With the exception of these two blocks and movement of two to four gene clusters, corresponding to either transposition or translocation of large DNA fragments, the remaining single gene shuffling events may correspond to random small-scale duplication (SSD).

Investigation of duplications (illustrated by the *Ks* peak <0.5, corresponding to <38 Ma) in grass evolution established that inter- and intrachromosomal single gene duplications exist independently from WGD events as random shuffling events. In *Brachypodium,* for example, SSD represents 38% interchromosomal duplications, 49% local tandem duplications, and 13% intrachromosomal duplications, as illustrated in supplementary figure S1, Supplementary Material online. Ancestral shuffled duplicates we identified (bottom circles of fig. 1*B* referenced as shuffled duplicates) could therefore correspond to ancestral SSD that took place 50–70 Ma. However, SSD implies the deletion of one of the duplicates generated by WGD, followed by duplication of the remaining copy, as we only considered gene pairs. SSD without deletion would lead to either three (post-WGD duplication) or four (pre-WGD duplication) copies. Nonsyntenic paleoduplicated genes may therefore be explained by either a single transposition event (first hypothesis) or successive deletion and duplication (second hypothesis) leading to SSD (supplementary fig. S2*A*, Supplementary Material online). To estimate whether SSD could explain all the observed cases of nonsyntenic paleoduplicates, we performed a complementary analysis on all the identified duplicates (supplementary fig. S2*B**,* top, Supplementary Material online) to separate syntenic paleoduplicates (supplementary fig. S2*B**,* middle, Supplementary Material online) from those that have not been retained on paralogous chromosomal fragments (supplementary fig. S2*B**,* bottom, Supplementary Material online). In this case, random SSD taking place in our WGD timing window would constitute a background level of duplicates, but we should not observe a *Ks* distribution peak, evidence of a unique event. The existence of the peak is in favor of our explanation that shuffled ancestral paralogs derive from a single transposition event (first hypothesis) involving only one copy of the ancestral pairs. A clear example is given in supplementary figure S2*C*, Supplementary Material online, of duplicated genes found as a conserved, WGD-derived pair in rice, whereas the orthologous genes in sorghum are not found in paralogous blocks. Thus, both WGD-based gene transposition/shuffling and ancestral random SSD are driving forces in gene movement. Finally, the shuffled duplicates corresponding to the ancestral WGD may be due to the transposition of one copy to a nonsyntenic location, but we cannot entirely exclude that ancestral SSD also contributed to this process. Such nonsyntenic ancestral duplicates not located in paleoparalogous blocks will thus be referenced as shuffled duplicates in the rest of the manuscript.

### Differential Retention of Genes and Functions in Duplicated Segments

**Fig. 2.—:**
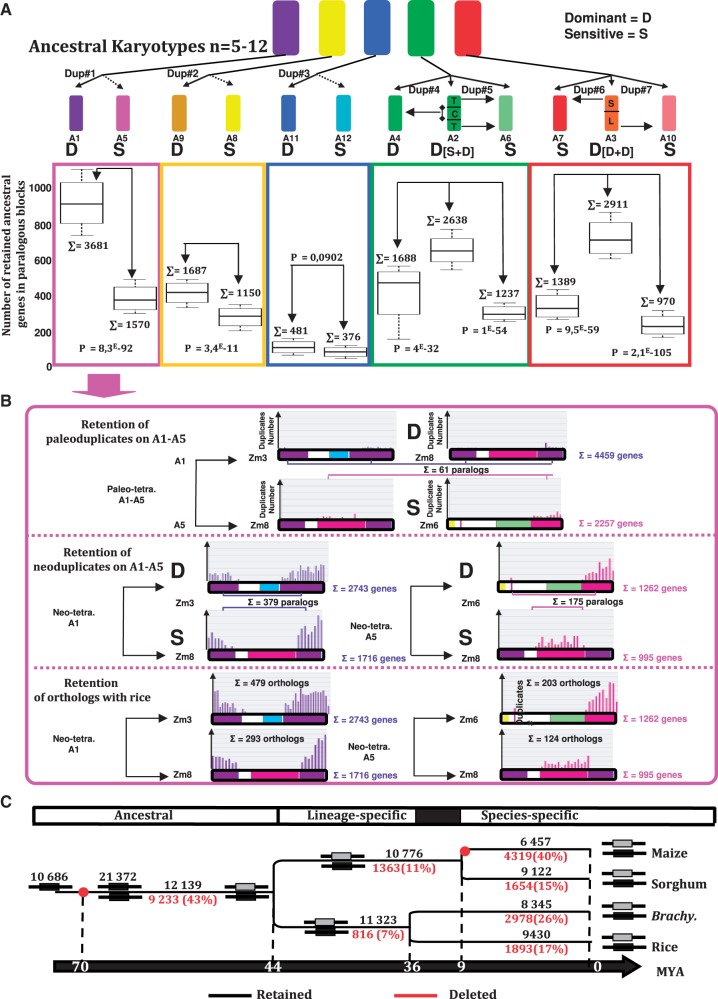
Grass subgenome partitioning. (*A*) Illustration of numbers of ancestral retained genes (ortholog repertoire) in paralogous blocks (*y* axis) observed in the modern genomes of *Brachypodium*, rice, sorghum, and maize derived from a single paleotetraploidy event from ancestral chromosomes A5, A8, A11, A4, and A7 (colored boxes). Mean and standard deviation values are shown. The statistical relevance is illustrated as *P* values based on paired χ^2^ test performed on retained (i.e., orthologous) gene numbers observed for each species (detailed in supplementary fig. S2, Supplementary Material online). Differences in gene content between paleoduplicated chromosomes are shown (**∑)** and define ancestral dominant (D) and sensitive (S) protochromosomes. Regarding the triplications A2-A4-A6 (green) and A3-A7-A10 (red), T = telomeric region, C = centromeric region, S = short arm, L = long arm. (*B*) Number of gene pairs characterized on maize chromosomes 3-6-8 deriving from protochromosome A5 between ancestral duplicates (illustrated at the top between purple and pink blocks), between recent duplicates (illustrated in the center between purple and pink blocks), and between maize/rice orthologs (illustrated at the bottom within purple and pink blocks), defining dominant (D) and sensitive (S) maize subgenomes. (*C*) Classification of the observed subgenome dominance as ancestral duplicate deletion (reported as total numbers and percentage of deleted duplicates in red) or retention (reported as total numbers of retained duplicates in black) in three classes: ancestral (left), lineage specific (middle), and species specific (right).

Bias in duplicated gene deletion has been described only in a few grass genomes or gene families (Schnable, Freeling, et al. 2012, Abrouk et al. 2012). We analyzed duplicated gene loss in four grass genomes to investigate subgenome dominance and determine whether subgenome partitioning is maintained in all species and whether it is an ancestral or recent process. By considering not only duplicated genes retained in their ancestral positions (colored circles [top] in fig. 1*B*) but also those that have been shuffled (colored circles [bottom] in fig. 1*B*), we observed that duplicated gene redundancy at the structural level is eroded by massive gene deletions and/or rearrangements (i.e., transposition or SSD). In maize, rice, *Brachypodium,* and sorghum, respectively, only 45‰, 32‰, 37‰, and 24‰ of genes are retained as duplicated, syntenic pairs, while 62%, 29%, 25%, and 40% of all ancestral retained duplicated genes are shuffled, cf. table 1*A*. However, these two phenomena are not random and appear more prevalent in one of the sister regions in all the species investigated (fig. 2*A*). On average, comparing orthologous blocks r1-b2-s3-m3-m8 (corresponding to A1) and r5-b2-s9-m6-m8 (corresponding to A5), 58% of genes are deleted in the former (among 17,937 genes with 7,466 retained genes), whereas 67% are deleted in the latter (among 10,411 genes with 3,478 retained). In supplementary figure S3, Supplementary Material online, statistical tests (see Materials and Methods), compare the ancestral retained gene content (the orthologous gene repertoire) between duplicated protochromosomes. Results of a χ^2^ test between observed and theoretical equal retention of duplicated genes are given in supplementary figure S3, Supplementary Material online, and reported as *P* values on figure 2*A*. Statistically significant differences in numbers of retained genes define dominant (D) and sensitive (S) paralogous blocks in modern grass genomes (except for the A11-A12 duplication, see next section), cf. figure 2*A*.

When considering the specific maize genome duplications as a model of recent WGDs, the subgenome partitioning is even more visible due to the recent nature of the tetraploidization event, that is, 5 Ma (table 1*B*). Figure 2*B* illustrates the acceleration of genome partitioning by successive rounds of WGDs in maize. Considering chromosomes derived from ancestral A1 and A5, the modern maize chromosomes m3–8 and m6–8 have 61 duplicates (i.e., conserved genes between purple light and dark blocks, first panel), whereas for the same paleotetraploidization event, r1-r5 (129), b2L-b2S (106), and s3-s9 (59) have 98 duplicates on average. However, when considering the neotetraploidization event that involved the same protochromosomes, corresponding to m3–8 and m6–8 in the modern maize genome (i.e., conserved genes between purple light blocks and between purple dark blocks, second panel), we observed 379 and 175 retained duplicates, respectively. The maize subgenome dominance pattern for the remaining four ancestral chromosomes (A4, A7, A8, A11) is presented in supplementary figure S4, Supplementary Material online. These observations suggest that during the last 5 Myr of evolution a similar gene shuffling rate is observed in maize as for the rice, *Brachypodium,* and sorghum ancestral shared paleopolyploidization event dating back to 50–70 Ma. Moreover, the 2-fold difference observed between the two recently duplicated fragments (i.e., 379 vs 175) supports our conclusion that the A5-derived chromosomes (purple blocks on m3-m6-m8 in fig. 2*B*) are still the sensitive chromosomal segments in the modern maize genome.

It appears clearly that dominant or sensitive orthologous chromosomes are conserved (i.e., orthologs) in the four modern grass genomes, defining an ancestral karyotype with ancestral dominant and sensitive protochromosome donors (fig. 2*A*, top). To precisely investigate whether the genome dominance is entirely ancestral (duplicate deletion before speciation) or still active in each species (duplicate deletion specific to modern sensitive blocks), we studied the chronology of the gene loss. Considering ancestral duplicates (colored circles [middle] in fig. 1*B*) for which at least one copy is conserved in another species, deletion patterns were classified as 1) ancestral (prespeciation), 2) lineage specific (postspeciation), or 3) species specific (supplementary fig. S5, Supplementary Material online). Duplicate deletions (43%) are observed as being ancestral (prespeciation). Therefore, analysis at the level of both the orthologous S and D genome segments and the genes is in favor of subgenome dominance initiated ancestrally and continued or even accelerated (in the case of maize) after speciation. This becomes lineage (11% for the PACCAD (maize/sorghum) clade and 7% for the BEP (rice/*Brachypodium*) clade) or species specific (40%, 15%, 26%, 17% specifically lost, respectively, in maize, sorghum, *Brachypodium*, and rice), cf. figure 2*C*.

### Exceptions in Subgenome Dominance Following Polyploidy

In addition to the general subgenome dominance phenomenon observed in all four grass genomes investigated and its acceleration with superimposed WGD events, biases exist depending on gene functions (supplementary fig. S6*A*, Supplementary Material online) or gene chromosomal locations (supplementary fig. S6*B*, Supplementary Material online). Supplementary figure S6*A*, Supplementary Material online, illustrates the diploidization-resistant genes that are also enriched in sensitive chromosomal fragments (i.e., maintained as duplicates after WGD and not lost in sensitive blocks) and diploidization sensitive genes enriched in dominant chromosomal fragments (i.e., retained as singletons after WGD after deletion in sensitive blocks). GO classifications for both molecular function and biological process were investigated for maize at the whole genome and paleoduplication levels, as well as for the neoduplication. The most enriched GO (*P* < 5%) at the molecular function level are “structural molecular activity,” “transporter activity,” “catalytic activity,” “electron carrier activity,” “molecular transducer activity,” “binding,” “enzyme regulator activity,” “antioxidant activity,” “nutrient reservoir activity,” and “TR activity.” We classified these ten GO classes as diploidization-resistant or -sensitive and observed that GO involved in regulatory processes (TR activity, enzyme regulator activity, and binding) exhibit the opposite response to diploidization with resistance for the recent duplication (i.e., maintained as duplicated pairs after the neo-WGDs) and sensitivity for the ancestral duplication (i.e., maintained as singletons after the paleo-WGDs). This observation may suggest that in plants the ancient retention of diploidization-resistant genes can be counterbalanced by the loss of duplicates for such functions in more recent WGD, and vise versa. This counterbalance retention of gene function in successive WGDs observed in plants is in contrast to mammalian diploidization-resistant regulatory genes that have be shown to be retained as pairs after each rounds of WGDs (supplementary figure S6*A*, Supplementary Material online, red dashed arrows), Murat et al. 2010. At the biological process level, “regulation of biological process” and “biological regulation” also show contrasting responses in the context of successive WGDs (supplementary fig. S6*A*, Supplementary Material online). It is also interesting to note that only the GO class “response to stimuli” remains sensitive to diploidization (and consequently to subgenome dominance) in both unique or successive WGDs, suggesting that this gene family/function (including disease-resistance genes) is shuffled constantly after WGDs, explaining the reported reduced level of gene conservation between species (supplementary fig. S6*A*, Supplementary Material online; Luo et al. 2012).

Several studies have already pointed out a highly conserved duplication in the subtelomeric region of chromosomes r11-r12 and orthologous regions of s5-s8 and b4, this conservation being due to recurrent gene conversion events (Jacquemin et al. 2009, 2011). Here, we have tested this hypothesis in the maize (chromosomes 1-2-3-4-10) and millet (chromosomes 8-3-7) genomes. Supplementary figure S6*B*, Supplementary Material online, clearly shows that this highly conserved duplication is located at orthologous positions in rice, sorghum, maize, *Brachypodium,* and millet and can thus be traced back to the shared ancestral tetraploidization 50–70 Ma. Interestingly, the structure of this region is different in each of the five species: a direct subtelomeric repeat in rice, interrupted by a long inversion repeat in sorghum, located on the same chromosome in *Brachypodium* through ancestral CF, reduplicated in maize and translocated from millet chromosome 3 to chromosome 7, although still in a subtelomeric location on chromosome 7. The locus is only structurally conserved with high duplicate conservation in modern species when the two sister regions are telomeric (i.e., rice, millet, and sorghum, although the conservation is lower in the proximal region of the latter due to inversion), whereas in maize and *Brachypodium*, where the orthologous blocks are pericentromeric in the present-day genomes, the conservation has been eroded (dating and *Ks* color code on the supplementary fig. S6*B*, Supplementary Material online). Finally, this region is directly involved in the chromosome differences observed between the phylogenetically related millet and sorghum genomes. Ancestral chromosome A12 (modern millet chromosome 3) has been broken precisely at the highly conserved telomeric regions and the derived fragments translocated to the modern millet chromosome 7. The evolution of this particular locus explains why statistically significant subgenome dominance was not detected between ancestral paralogous protochromosomes A11 and A12 (see fig. 2*A* and supplementary fig. S3*B*, Supplementary Material online).

Overall, both gene functions (i.e., TFs and TRs for older duplications and response to stimuli functions for recent duplications) and chromosomal locations (i.e., gene conversion-based retention near the telomere) can locally reduce the previously reported diploidization-derived subgenome dominance phenomenon established at the whole genome level.

### Mechanisms Driving Gene Shuffling between Subgenome Compartments

**Fig. 3.—:**
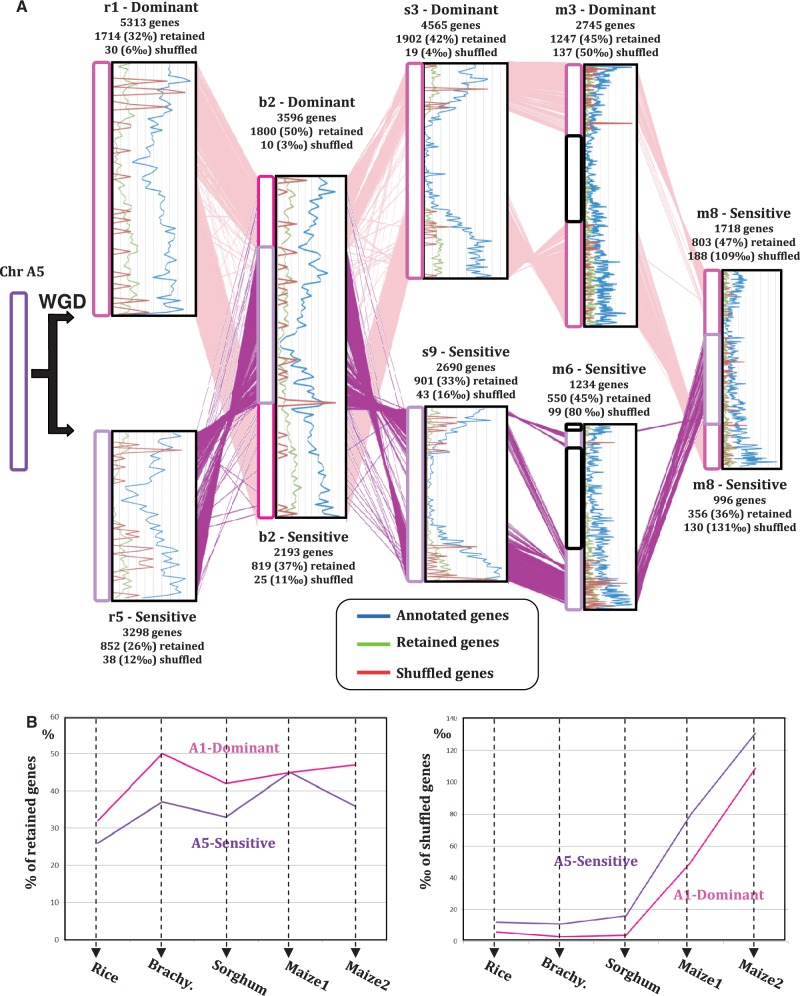
Gene conservation and movement in dominant and sensitive subgenomes. (A) Illustration of paralogous chromosomes in rice (Chr 1-5), *Brachypodium* (Chr 2), sorghum (Chr 3-9), and maize (Chr 3-6-8), originating from a single ancestral preduplication chromosome A5 (left). The orthologous genes are illustrated with colored lines between chromosomes. The distribution of annotated genes (blue curve), ancestral retained paralogous genes (green curve), and shuffled duplicated genes (red curve) are shown at the right side of the chromosomes. The total numbers of annotated, retained (i.e., ortholog), and shuffled genes are shown for each chromosome (top) defining dominant (referenced as D) and sensitive (referenced as S) chromosomal segments. (*B*) Graphic representation of the observed rate of retained (left) and shuffled (right) genes for the dominant (blue curve) and sensitive (red curved) orthologous chromosomes in rice, *Brachypodium*, sorghum, and maize (two paralogous regions deriving from the recent and specific WGD) deriving from the ancestral protochromosome A5.

We mapped the duplicated genes not located in paleoparalogous blocks (referenced as shuffled) in dominant and sensitive subgenomes on the basis of retained versus deleted duplicates. Figure 3*A* illustrates the gene-shuffling frequency between dominant and sensitive subgenomes in the modern grass chromosomes derived from protochromosome A5 (i.e., rice chromosomes 1–5, *Brachypodium* chromosome 2, sorghum chromosomes 3–9, and maize chromosomes 3-6-8). Annotated genes (blue curves), ancestral retained genes (green curves), and shuffled genes (red curves) have been mapped on these modern chromosomes. The distribution of conserved or syntenic genes follows the known distribution of genes along chromosomes, with high density in subtelomeric regions and low density in centromeric ones (Murat et al. 2010). Figure 3*B* shows that lower gene retention (with an average of 43% of ancestral retained genes in dominant genomes vs. 35% in sensitive ones) and higher gene movement (with an average 34‰ of shuffled genes in dominant genomes vs. 50‰ in sensitive ones) are observed in dominant and sensitive chromosomes deriving from the ancestral chromosome A5 in all four species (including two regions in maize). This suggests that genome sensitivity is driven by lower gene conservation, involving both gene deletion and movement (duplicate transposition or, to a lesser extent, ancient SSD). In this scenario, subgenome dominance is driven either by 1) massive loss of genes from the S compartment, 2) transposition of genes from the S compartment, or 3) SSD, via gene deletion in the D compartment and duplication of the sister copy from the S compartments (supplementary fig. 2*A*, Supplementary Material online).

We then investigated the structures and functions of duplicated genes not located in paleoparalogous blocks. In rice, considered to be the closest representative of the *n* = 12 grass ancestor, these duplicates have reduced numbers of exons (on average 4.6/gene, supplementary fig. S7*A*, Supplementary Material online) and are shorter (on average 2,940 bp, supplementary fig. S7*B*, Supplementary Material online), compared with all rice-annotated genes (on average 4,728 bp structured in 8.6 exons). This raises the possibility that shuffled duplicates could be pseudogenes (Wicker et al. 2011; Yang et al. 2011). We also investigated the GO classification and observed that conserved duplicates are enriched (*P* < 5%) for functions corresponding to “binding activity,” in contrast to shuffled duplicate genes, which are enriched for “biological activity” (supplementary fig. S7*C*, Supplementary Material online). This is consistent with reported diploidization-resistant gene functions discussed above, such as TFs and TRs, belonging to the binding activity category. Finally, duplicate genes not located in paleoparalogous blocks also appear to show reduced expression compared to conserved genes (expressed genes are associated with at least one cognate expressed sequence tag [EST], supplementary fig. S7*D*, Supplementary Material online).

**Fig. 4.—:**
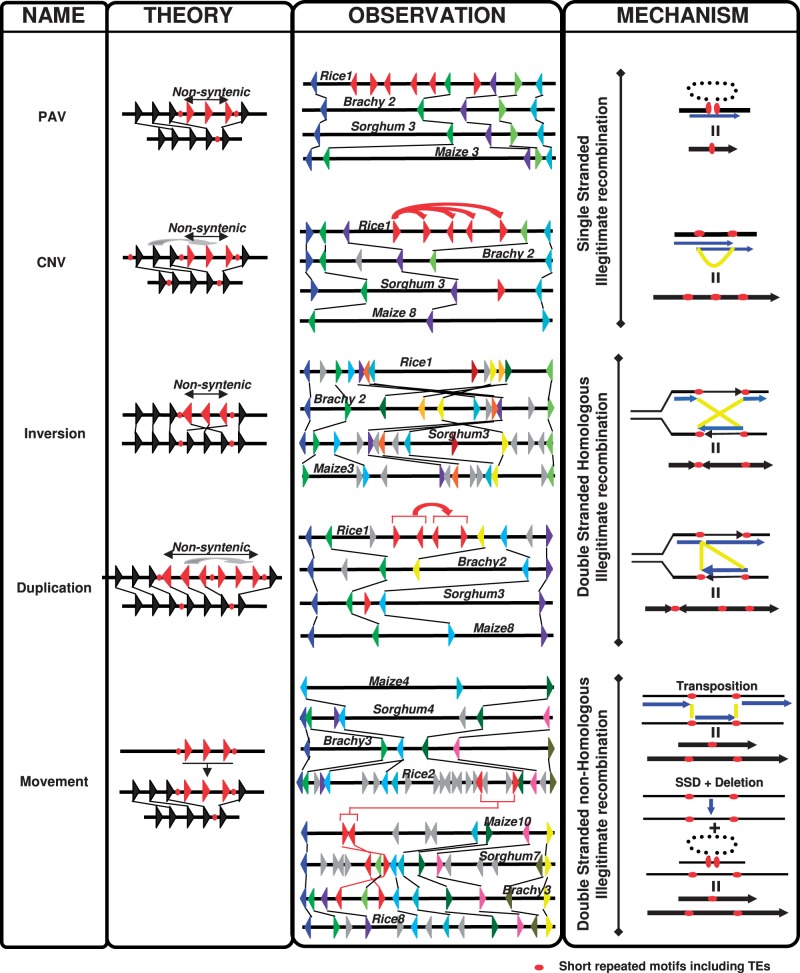
Putative hypothetical molecular mechanisms driving nonsyntenic ancestral genes in grasses. PAV of LOC_Os01g07640, LOC_Os01g07650, LOC_Os01g07660, LOC_Os01g07670, LOC_Os01g07680, and LOC_Os01g07710 is illustrated between rice chromosome 1 (chromosome position 3.6 Mb, containing 11 genes with 51 kb physical size), *Brachypodium* chromosome 2 (position 3.1 Mb, 5 genes, 29 kb), sorghum chromosome 3 (position 4.6 Mb, 5 genes, 49 kb), and maize chromosome 3 (position 12 Mb, 5 genes, 144 kb). CNV of LOC_Os01g16360, LOC_Os01g16370, LOC_Os01g16380, LOC_Os01g16390, and LOC_Os01g16400 is illustrated between rice chromosome 1 (position 9.3 Mb, 10 genes, 64 kb), *Brachypodium* chromosome 2 (position 8.3 Mb, 6 genes, 61 kb), sorghum chromosome 3 (position 1.6 Mb, 5 genes, 20 kb), and maize chromosome 8 (position 3.4 Mb, 3 genes, 69 kb). Inversion between LOC_Os01g01170 and LOC_Os01g01307 is illustrated between rice chromosome 1 (position 0.1 Mb, 13 genes, 107 kb), *Brachypodium* chromosome 2 (position 2.6 Mb, 11 genes, 51 kb), sorghum chromosome 3 (position 9.8 Mb, 15 genes, 120 kb), and maize chromosome 3 (position 32 Mb, 13 genes, 717 kb). Duplication for LOC_Os01g16370, LOC_Os01g16380, LOC_Os01g16390, and LOC_Os01g16400 is illustrated between rice chromosome 1 (position 9.2 Mb, 11 genes, 74 kb), *Brachypodium* chromosome 2 (position 8.2 Mb, 6 genes, 65 kb), sorghum chromosome 3 (position 11.7 Mb, 5 genes, 51 kb), and maize chromosome 8 (position 3.4 Mb, 4 genes, 116 kb). Movement of LOC_Os02g19130 and LOC_Os02g19060 is illustrated between rice chromosome 8 (position 3 Mb, 13 genes, 92 kb), *Brachypodium* chromosome 3 (position 15.1 Mb, 12 genes, 94 kb), sorghum chromosome 7 (position 4.1 Mb, 17 genes, 227 kb), and maize chromosome 10 (position 80.2 Mb, 10 genes, 453 kb). The hypothetical molecular processes (first column) driving nonsyntenic genes in grasses are illustrated through theoretical (second column) versus real (third column) examples (detailed previously) and associated DNA mechanisms involving either single versus double-stranded or illegitimate versus homologous recombination (fourth column).

When comparing genes between species, nonconserved duplicates have been deleted (referenced hereafter as PAV for presence/absence variation between species), duplicated in tandem (CNV for copy number variation between species), inverted, duplicated within a block or moved (transposition of one copy or SSD consisting in deletion of one copy and duplication of the other) in the course of evolution. As described earlier, we concluded that duplicated gene loss is mainly due to deletion (an average of 35‰ of duplicates are retained between duplicated blocks) and the other nonsyntenic retained duplicates lead to CNVs, inversions, duplications, and, more generally, gene movement. In figure 4, we propose molecular mechanisms at the DNA level that may have driven such loss in genome synteny, refining mechanisms previously proposed in the literature (Bzymek et al. 1999; Chantret et al. 2005; Wicker et al. 2010; Woodhouse, Pedersen, et al. 2010). Precise examples of PAVs, CNVs, inversion, duplication, and movement (i.e., transposition) characterized in grasses are given.

PAVs and CNVs may be explained by single-strand DNA illegitimate recombination shuffling mechanisms. The PAV example (1 in fig. 4) involves a noncollinear gene cluster in rice (highlighted in red) that can be modeled by a segmental deletion during replication of a DNA loop formed by illegitimate recombination involving short sequence/repeat motifs (red dots). Such ancestral motifs are no longer detectable in modern intergenic regions due to high nested TE turnover. We suggest that this DNA shuffling event took place in the Pooideae/Panicoideae ancestor, making this deletion detectable in the rice genome as the representative of the Ehrhartoideae. The CNV example (2 in fig. 4), involving a noncollinear gene cluster of tandem duplicates in rice (highlighted in red), can be modeled by local duplication of genes by replication slippage using short sequence/repeat motifs (red dots) as a matrix. We suggest that this DNA shuffling event took place during the last 30 Myr of evolution, specifically in the Ehrhartoideae lineage so that it is not detectable in modern Pooideae or Panicoideae species.

The inversion (3 in fig. 4), duplication (4 in fig. 4), and movement (5 in fig. 4) mechanisms may involve homologous DNA strand exchange through double-stranded DNA illegitimate recombination. It is possible to assume that short intergenic repeat motifs (red dots) may have favored large inversions by complementary DNA strand exchange during replication, as illustrated (3 in fig. 4) with a chromosomal segment inverted on the *Brachypodium* region compared with rice, sorghum, and maize. Segmental duplication in similar or inverted orientation can also be modeled through double-strand break (DSB) repair or replication slippage, as illustrated (4 in fig. 4) for a cluster of two tandem genes in rice that has been duplicated locally (highlighted in red), with the same relative orientation.

Finally, the gene and/or genomic block movement or transposition may involve nonhomologous DNA strand exchange through double-strand DNA illegitimate recombination shuffling mechanisms. In the gene movement example (5 in fig. 4), the rice orthologous genes (highlighted in red with rice chromosome 3 as donor region), conserved between chromosomes 3, 7, and 10 in *Brachypodium,* sorghum and maize, respectively, were transposed to rice chromosome 2 (acceptor region) at a nonorthologous position and are thus absent from the orthologous region on chromosomes 3, 4, and 4 in the other three species. Alternatively, a less parsimonious scenario would consist in the SSD of the gene from the donor to the acceptor region followed by the deletion of the gene from the donor site. Overall, all these major grass genome shuffling events can be modeled through either single- versus double-stranded or illegitimate versus homologous DNA recombination mechanisms.

### Revisiting the Monocot Ancestors Based on Chromosomal Partitioning

**Fig. 5.—:**
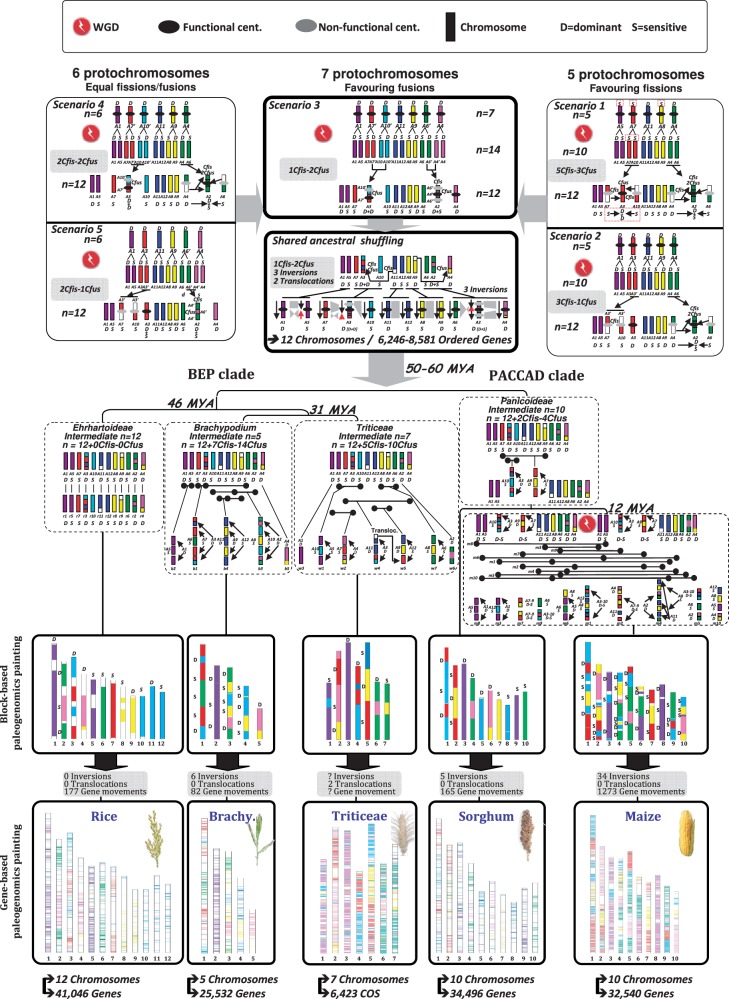
Evolutionary model of the grass genome deriving from a *n* = 5 to 7 ancestor. The modern monocot chromosomes (bottom) are represented with color codes to illustrate the evolution of segments from a common ancestor with five-six-seven protochromosomes (named according to the rice nomenclature from 1 to 12) as detailed in the text in five scenarios (top). The four shuffling events that have shaped the structure of the different grass genomes during their evolution from the common ancestor are indicated as WGD (red dots), ancestral chromosome translocations and fusions (black/red arrows), family specific as well as lineage-specific shuffling events (referenced as inversions, translocations, and gene movements). WGD, functional and nonfunctional centromeres (black and gray dots, respectively), dominant (D) and sensitive (S) subgenomes are illustrated according to the figure legend shown at the top. The structure of the modern genomes is represented at the bottom of the figure (number of chromosomes and genes, or COS for wheat, are referenced) as chimera of paleo-subgenomic regions at the large chromosomal segment level (i.e., referenced as block-based paleogenomics painting) or locus level (i.e., referenced as gene-based paleogenomics painting).

The precise characterization of ancestral duplicates that are maintained at syntenic locations in modern genomes and of duplicates that have been deleted/shuffled, driving subgenome dominance, allowed us to precisely identify dominant (D) and sensitive (S) subgenomes in modern grasses and reinvestigate the proposed evolutionary scenario from a founder ancestral karyotypes of five to seven protochromosomes (recently reviewed in Salse 2012 and Murat et al. 2012). The analysis of the fate of ancestral duplicates clearly established that in the *n* = 12 ancestral intermediate, A1-2-3-4-9-11 are dominant segments and A5-6-7-8-10-12 are sensitive ones (cf. fig. 2*A*). Moreover, the precise identification of ancestral genes that are still retained in modern genomes but at nonorthologous positions defined two previously unreported ancestral duplications involving A3-A12 and A4-A8 (cf. fig. 1*B* blue and yellow duplications within bottom circles). Based on these findings, we revisited the paleoevolutionary scenario we recently proposed, which is illustrated in figure 5 (top, scenarios 1–5; Salse 2012).

In scenario 1 (fig. 2*A,* top), we proposed (Salse et al. 2008, Salse, Abrouk, Bolot, et al. 2009) that an *n* = 5 (A4/A5/A7/A8/A11) ancestor was duplicated to reach an *n* = 10 intermediate. A5(S) and A8(S) are duplicated into A1 and A9, respectively, that become dominant after the WGD, and A11(D) is duplicated into A12(S). Although this evolution involves no additional event since the ancestral WGD, the question still remains open on the precise origin of modern chromosomes A2-A3-A4-A6-A7-A10. We suggested that, after shared paleotetraploidization, additional fissions and fusions took place on these protochromosomes to reach the *n* = 12 ancestor common to the modern monocot genomes. These specific shuffling events should explain the two triplications found in any modern monocot genome and involving, for example, rice r2-r4-r6 and r3-r7-r10. In scenario 1, the shared triplication A3(D)-A7(S)-A10(S) originated from a single A7(S) protochromosome duplicated into the A10(S) protochromosome. We suggested that both A7(S) and A10(S) were split by a centromeric break and A7L (L for long arm) was fused to A10S (S for short arm), forming A3(D). The remaining A7S and A10L protochromosomes harbor nonfunctional centromeres (partially deleted due to the proposed fission events), illustrated as gray dots in figure 5. Taking into account subgenome dominance, this scenario implies the formation of dominant chromosome A3 as a fusion of sensitive A7–A10 protochromosomes. We can consider that 1) a sensitive ancestral protochromosome and 2) the fusion of two sensitives, leading to a dominant protochromosome (S + S ≠ D) are not possible. Similarly, the shared triplication, A2-A4-A6, originating from a single A4(D) protochromosome duplicated into A6(S), would have arisen from a fusion between A4 and A6 to form A2(D), leaving A4 and A6 with nonfunctional centromeres. In scenario 1, three of the five proposed protochromosomes (A5, A7, and A8) are sensitive and produce modern dominant chromosomes, respectively, A1, A3, and A9 (with higher ancestral gene content), after WGD. This is, by definition, impossible as an ancestral S chromosome cannot recover dominance by neoaccumulation of paleodeleted orthologous genes. Overall, taking into account subgenome dominance and centromere functionality, this scenario is rejected.

We investigated four new alternative scenarios (fig. 5, scenarios 2–5, top) based on the following assumptions. 1) fusions involve both dominant and sensitive chromosomes. Fusion of two S chromosomes cannot give a D chromosome (S + S ≠ D) and fusion of two D chromosomes cannot give an S chromosome (D + D ≠ S). There is a hierarchy in dominance/sensitivity fusion, such that D + D = D > D + S = D > S + S = S. 2) the derived ancestral chromosomes should harbor functional centromeres after fusion and fission events. Figure 5, scenario 2, is still based on an *n* = 5 ancestor composed of the identified dominant protochromosomes (A1-A3-A4-A9-A11). A1, A9, and A11 lead to pairs of S and D chromosomes after WGD with, respectively, A1(D)**→**A5(S), A9(D)**→**A8(S), and A11(D)**→**A12(S), and the remaining A2-A3-A4-A6-A7-A10 chromosomes derive mainly from fission events. We propose that A3(D) was duplicated into an A3′ ancestral chromosome that became sensitive and then split into A7 (corresponding to A3′L) and A10 (corresponding to A3′S). Although the subgenome dominance observed for A3-A7-A10 in this scenario is in agreement with that observed in modern grasses (fig. 2*A* with A3 = D, A10 = S, and A7 = S), the proposed A7 and A10 protochromosomes both carry nonfunctional centromeres (gray dots, fig. 5). The same mechanism favoring fission events can also be proposed for the origin of A2-A4-A6 protochromosomes as illustrated in figure 5, leading to the same incongruency regarding centromere functionality (as gray dots). Overall, both scenarios deriving from an *n* = 5 ancestor can be rejected, because of either the subgenome dominance rules (scenario 2) or the centromere functionality rule (scenario 1 and 2).

In figure 5, scenario 3, based on an *n* = 7 ancestor, dominance rules are consistent with the previous scenario for A1, A9, A11 as dominant ancestral chromosomes and the derived duplicated A5(S), A8(S), and A12(S) chromosomes, respectively. For the remaining protochromosomes, we performed detailed analysis in rice of the A4-A2, A6-A2, A10-A3, A7-A3 paralogous regions (cf. diagonals of the dot plot illustrated as supplementary fig. S8, Supplementary Material online). Based on the orthologous gene repertoire of such regions defining dominance and sensitivity, we established that A6 = S (1,237 retained orthologs characterized in grasses), A4 = D (1,688 orthologs), A7 = D + S (with 1,316 orthologs for the sister fragment of A6 and 1,322 orthologs for the sister fragment of A4). Similarly, A10 = S (970 retained orthologs characterized in grasses), A7 = S (1,389 orthologs), A3 = D + D (with 1,027 orthologs for the sister fragment of A10 and 1,884 orthologs for the sister fragment of A7), supplementary figure S8, Supplementary Material online. In this scenario, the triplications A2-A4-A6 and A3-A7-A10 each derive from two distinct ancestral chromosomes. For A3-A7-A10, dominant ancestral chromosomes A10′ and A7′ are duplicated into A10 and A7, respectively, that became sensitive and are characterized as such in all modern genomes (fig. 2*A*). A3 derives from the telomeric fusion (also referenced as TCF for telomeric CF, supplementary fig. S8, Supplementary Material online) of A10′ (D) and A7′ (D) so that these two protochromosomes are no longer identified separately in any modern genome. In this scenario, dominant chromosome A3 results from A10′ (D) + A7′ (D), that is, D + D = D. Similarly, in the case of A2-A4-A6, dominant ancestral chromosomes A6′ and A4 are duplicated into A6 and A4′, respectively, that became sensitive and are still characterized as such in all modern genomes (fig. 2*A*). We suggest that A2 (D) derived from the centromeric fusion (also referenced as CCF for centromeric CF, supplementary fig. S8, Supplementary Material online) of A6′ and A4′. In this hypothesis, A2 (D resulting from D + S, fig. 2*A*) = A4′ (S) + A6′ (D), that is, S + D = D. This scenario necessitates only one fission and two fusions (figure 5, top center), compared with five fissions and three fusions in the *n* = 5 scenario. The other two alternative scenarios, combinations of the *n* = 5 and *n* = 7 scenarios, lead to a putative *n* = 6 ancestor (fig. 5, scenario 4–5, top). For either the A2-A4-A6 (scenario 4) or A3-A7-A10 (scenario 5) chromosome groups, both favor the fission hypothesis (initially considered for the *n* = 5 ancestors in scenarios 1–2) but lead to incongruency for either the subgenome dominance or centromere integrity rules. The illustrated scenarios, implying an ancestral structure of *n* = 6 protochromosomes, are thus rejected. In conclusion, the revised evolutionary model based on an *n* = 7 scenario is more parsimonious (less fusion and fission events) and consistent with segmental dominance/sensitivity and centromere functionality observed in present-day grass genomes (as illustrated for the rice genome in supplementary fig. S9, Supplementary Material online). This definitively establishes rice as the closest relative of the *n* = 12 ancestral karyotype structure.

### Revisiting Monocot Paleohistory from the *n* = 12 Ancestor

We have refined the evolutionary scenario for the investigated lineages (and not only the ancestral chromosome structure of *n* = 5–7) based on reanalysis of syntenic and nonsyntenic duplicated genes as well as taking into account the fate of ancestral D and S compartments. Based on the Blast-derived orthologus relationships (defining 16,464 protogenes) and using Cynteny/DRIMM-synteny to define syntenic groups and ANGES to define ancestral gene order in these groups, the BEP and PACCAD clades derived from the *n* = 12 chromosomes contain 6,246 ordered protogenes taking into account all the investigated species and 8,581 excluding the recently duplicated maize genome (see Materials and Methods and supplementary fig. S10, Supplementary Material online). We then identified ancestral shuffling events that took place between the duplicated blocks in the *n* = 12 ancestor with three ancestral inversions (in A5/A3 and A2 protochomosomes) as well as two ancestral translocations (between A4-A8 and A3-A12 protochromosomes), as illustrated with red and black arrows in figure 5 (top middle panel). The modern grass genomes have evolved from this ancestral genome structure through independent CCF, TCF events, inversion, translocation, and gene movement to reach their modern known karyotypes.

The modern rice genome has retained the original chromosome number of 12, derived from the postduplication *n* = 12 ancestral intermediate (supplementary fig. S11, Supplementary Material online), with no lineage-specific CCF or TCF but 177 characterized gene movements (fig. 5). *Brachypodium* went through seven CCFs (four involving the fusion of D + S, two and one involving, respectively, S + S, D + D protochromosomes, supplementary fig. S12, Supplementary Material online), highlighted with distinct colors and shown with black arrows on the same chromosomes, as well as six chromosomal inversions (CIs) and 82 gene movements (fig. 5). The ancestral maize and sorghum genomes evolved from the 12 intermediate ancestral chromosomes through two CCF (between A3(D) and A10(S); A7(S) and A9(D), fig. 5), giving the progenitor genome of the Panicoideae ancestor with *n* = 10 (12–2) chromosomes. Maize and sorghum subsequently evolved independently, with the sorghum genome maintaining the *n* = 10 structure of the ancestral genome except for 5 CIs and at least 165 specific gene movements (fig. 5), while maize underwent another WGD event, resulting in an intermediate with *n* = 20 chromosomes. Rapidly following this event, 7 CCFs + 10 TCFs (supplementary fig. S12, Supplementary Material online) led to a genome structure with ten chromosomes followed by 34 CIs and 1,273 gene movements. Overall, a minimum of 71 large-scale and 2,067 gene-based rearrangement events (16 CCFs [7 for *Brachypodium*, 2 for sorghum, and 7 for maize], 10 TCFs [maize only], 45 CIs [6 for *Brachypodium*, 5 for sorghum, and 34 for maize], 2,067 gene shufflings [177 for rice, 82 for *Brachypodium*, 135 for sorghum, and 1,673 for maize]) took place during the last 50–70 Myr of evolution to shape the modern grass genome architecture from the reconstructed *n* = 12 founder ancestor. The wheat syntenome published recently (Salse et al. 2008, Pont et al. 2013) has been used to integrate the Triticeae, represented by wheat, into this grass evolutionary scenario, even though the sequence is not available. The Triticeae genomes derived from the *n* = 12 ancestor through five CCFs involving, with (w) for wheat, w1(S) = A10(S) + A5(S), w2(D) = A7(S) + A4(D), w3(D) = A1(D), w4(D) = A11(D) + A3(D), w5(D) = A9(D) + A12(S), w6(D) = A2(D), w7(S) = A8(S) + A6(S), figure 5. The Triticeae went through additional lineage-specific events, among which we can only identify the large-scale ones, including two translocations we precisely located between w4-w5-w7 (A4-A5 translocation shared by all the Triticeae and A4-A7 specific to wheat).

## Discussion

### A Polyploidization-Based Diploidization Process Drives Grass Genome Plasticity

Most comparative genomics studies rely on the characterization of groups of genes that are retained at ancestral (i.e., conserved) positions, either between modern species (synteny analysis) or within modern species (duplication analysis). Here, we have considered ancestral genes (16,464 protogenes, either conserved between orthologous and paralogous regions in grasses), taking into account not only retained pairs located within known orthologous or paralogous blocks but also those that have been deleted or shuffled. This last class may have been either transposed from the previous donor regions to a new acceptor location or ancestrally duplicated (SSD) from the donor to the acceptor region followed by the deletion of the donor site. This allowed us to precisely identify deleted and shuffled ancestral duplicated genes as the processes driving paleopolyploid genome diploidization.

DNA recombination has been suggested as the main cause of observed gene deletion or shuffling events. Both homologous and illegitimate DNA recombination processes involve pairing of two copies of short repeats, with the extent of similarity between such repeats and the exact mechanisms involved being quite different (Bzymek et al. 1999; Chantret et al. 2005; Wicker et al. 2010; Woodhouse, Pedersen, et al. 2010). In contrast with homologous recombination, illegitimate recombination events require only limited and smaller sequence motifs and occur in any region, eventually removing all unselected sequences (Kirik et al. 2000; Devos et al. 2002). Our results suggest that rejoining/deletion/transposition/duplication of DNA fragments of several kilobases in length, either on single- or double-strand DNA (between homologous as well as nonhomologous segments) may have required only a few base pairs of conserved sequence (Gorbunova and Levy 1999). This could explain the variable distribution, size, and sequence composition of the deleted and shuffled DNA fragments, leading to the described nonsyntenic genes identified between grasses (referenced as PAV, CNV, inversion, duplication, and transposition shuffling events).

It has been shown that duplication events in grass paleohistory have been followed by structural partitioning, defining postduplication-dominant regions (structurally stable with higher retention of protogenes) in contrast to sensitive paralogous counterparts (structurally plastic with higher loss of protogenes). Duplicated gene deletion and movement following WGD, which account for a large part of plant genome plasticity, are not random at the genome, chromosome, or gene function levels. We observed that these processes occur at a higher rate in sensitive chromosomal compartments, suggesting that subgenome dominance originates from biased duplicated gene deletion and movement between sister blocks. The in-depth characterization of dominant or sensitive chromosomal compartments in the four grasses is in favor of subgenome dominance initiated ancestrally (probably immediately after WGD with 43% of ancestral duplicates lost before speciation) and continued or even accelerated (in the case of maize with superimposed rounds of WGD) after speciation. Overall, these data suggest that DNA rearrangements at the chromosome and gene levels, leading to this diploidization-driven subgenome dominance, occurred immediately after polyploidization, probably within a few generations.

Duplicated gene shuffling (i.e., ancestral duplicate deletion, transposition, or SSD, all explaining the observed D/S blocks partitioning) involved a particular class of genes with short size, reduced number of exons, and particular functions (TFs and TRs for paleoduplications and response to stimuli for neoduplication events). This particular typology raises the question of pseudogenes as major candidates for mobile genes. The most complete and recent study characterizing pseudogenes was performed in *Arabidopsis*, showing that they are shorter and less expressed (Yang et al. 2011), which is also the case for shuffled genes in grasses. The identification of mobile genes with reduced size, and fewer exons, suggests that they were not transposed as full copies but putatively as 5′ or 3′ truncated fragments. Moreover, the identification of fewer perfect matches in EST databases for the shuffled genes (24%) compared with the conserved genes (48%) is in favor of transposed genes being pseudogenes known to be less expressed. We conclude that transposed and deleted duplicated genes account, to a great extent, for the reported subgenome dominance in paleopolyploid grasses (Abrouk et al. 2012). This raises the hypothesis that underexpressed genes in sensitive subgenomes are more likely to become deleted or transposed, leading to long-term pseudogenization, a process defining subgenome dominance, because these genes may be less important for maintenance of a perfect gene product balance and are thus less essential for fitness (Freeling et al. 2012).

Overall, gene deletion or movement may appear as a particularly active phenomenon after polyploidy and may then act preferentially on the sensitive genome compartments, making them more labile than the orthologous dominant counterpart. The proposed impact of polyploidization-based subgenome partitioning on contrasted gene content and diversity in dominant and sensitive blocks may need to be reconsidered in phenotype or even trait investigation in grasses. It has been suggested that visible phenotypical changes resulting from differential gene expression and/or knockouts will depend on the dominant or sensitive nature of the targeted genomic regions (Wang et al. 2009; Schnable and Freeling et al. 2011). Indeed, the labile, sensitive genomic compartment may carry gene copies that can be co-opted from ancestral to innovative or adaptive function/expression patterns that are more species specific (Roulin et al. 2012). The current study now opens a new paradigm that still needs to be proven, where grass adaptation (in particular, adaptation in response to biotic and abiotic stresses) may possibly have been partitioned between the currently defined dominant and sensitive chromosomal compartments in the genome.

### Grass Paleohistory Follows Precise Evolutionary Rules Revealing an Ancestor of *n* = 7 and 16K Protogenes

The detailed characterization of conserved, deleted, and shuffled duplicates allowed us to unravel chromosome dominance for the investigated grass genomes. Although genome portioning following polyploidy has been proposed to be a pure postspeciation or even lineage/species-specific process (Schnable, Freeling, et al. 2012; Schnable, Wang, et al. 2012), our observations at both genome and gene levels suggest that almost half of the deleted ancestral duplicated gene copies are common to modern species, and this deletion is therefore ancestral or prespeciation. We provide here a complete picture of subgenome dominance and sensitivity that allowed us to define new rules that drive the evolution toward modern species. Rule 1: Polyploidization drives dominant (D) and sensitive (S) subgenomes. The ancestral shared WGD defines a precise set of dominant and sensitive subgenomes that may condition their potential fusion patterns. Rule 2: Subgenome dominance is mediated by both gene deletions and gene shuffling from the S blocks. Rule 3: Gene deletion/movement may be mediated by single-/double-stranded illegitimate recombinations. Rule 4: Modern species derived from the centromeric fusion of protochromosomes leading to functional monocentric neochromosomes. Rule 5: The fusion of two dominant blocks led to a supradominant neochromosomes (D + D = D) with higher ancestral gene retention compared with D + S = D or even S + S = S.

By providing a complete picture of subgenome dominance and sensitivity in modern and ancestral grasses following these evolutionary rules, we propose a robust revised evolutionary model from an *n* = 7 ancestor (containing 16,464 protogenes with up to 8,581 ancestrally ordered, based on conserved gene adjencies between modern grasses). In this scenario, the seven proposed protochromosomes are dominant and went through a paleopolyploidization event to reach an *n* = 14 intermediate, followed by one chromosome fission, two fusions, and three inversions that shaped the *n* = 12 ancestral intermediate. We cannot exclude that the investigated bias in gene context/expression reflecting ancestral subgenome dominance in grasses may be evidence that the pregrass duplication resulted from an allotetraploidy event between ancestral parent 1 (A4′-A5-A6-A7-A9-A10-A11) and ancestral parent 2 (A4-A1-A6′-A7′-A8-A10′-A12). This is the only scenario that fits with the rules defined above and explains how modern grass karyotypes have been shaped by a unique founder preduplication ancestor of *n* = 7 (with 1,148 ordered protogenes) and a postduplication *n* = 12 (with 6,246 ordered protogenes taking into account all investigated species or 8,581 excluding maize), followed by a minimum of 71 large (inversions, translocations) and 2,067 small lineage-specific shuffling (movement such as transposition and SSD) events.

Our high-resolution evolutionary study clarifies open questions regarding specific chromosomal regions that have long been studied in monocots. There have been numerous speculations on the observed reduced density of genes on the short arms of chromosome 5-4-6-1, respectively, in *Brachypodium*, rice, sorghum, and wheat (International *Brachypodium* Initiative 2010). It has been suggested that such TE-rich regions were established early in evolution as “nesting grounds for repeats” (Wicker et al. 2011). This is probably not the case as we clearly established that these regions were translocated early during grass evolution, and the “missing” short arms of these chromosomes (pretranslocation chromosomes 4-9-2-5) have been transposed to the modern (posttranslocation) chromosomes 5-4-6-2, respectively, in *Brachypodium*, rice, sorghum, and wheat. In fact, these chromosomal structures are absolutely normal in terms of repeats and gene densities but split over two chromosomal regions in the present-day genomes. The high rate of conservation observed between rice chromosomes 11 and 12 has also long been a source of speculation (Murat et al. 2010; Wang et al. 2011). Although most of the paleoduplicated blocks evolved following the subgenome dominance rules derived from targeted gene deletion and transposition on the sensitive subgenomes, one ancestral pair of duplicated chromosomes evolved in an exceptional manner. The addition of foxtail millet and maize in this study not only extends and confirms this observation, but also demonstrates the strong influence of chromosome position and structure on evolution of this region. A high degree of gene conservation is observed in rice, (11–12), sorghum (5–8), *Brachypodium* (4L-4S), foxtail millet (7–8), and maize (1-2-3-4-10). Subgenome dominance at these loci has itself been dominated by a concerted evolution process. Gene conversion (for review, Marais 2003) between the duplicated blocks may account for a large part of such observed conservation for these regions. However, this process was initiated in the grass ancestor as the orthologous regions are highly conserved in all the modern genomes investigated and has been shown to be recurrent, at least in the Oryza genus (Jacquemin et al. 2009, 2011). Differences in conservation rate in modern genomes is observed depending on whether the regions remained telomeric (high conversion, such as in rice, millet, and sorghum) or became pericentromeric (conversion eroded, such as in *Brachypodium* and maize) following evolutionary shuffling events such as ancestral CFs. Such observed bias in gene conversion associated with extensive homeologous gene loss is the signature of sex chromosomes in human (Lahn and Page 1999), fungi (Charlesworth 2002), and plants (Ming et al. 2007). This hypothesis opens the question of ancestral chromosome 11 (A11) as a putative close relative of the sex chromosomes in mammals (Wang et al. 2011).

### Updated Crop Circles Based on a New Public “PlantSyntenyViewer” Tool Can Be Used as a Guide for Translational Genomics in Grasses

**Fig. 6.—:**
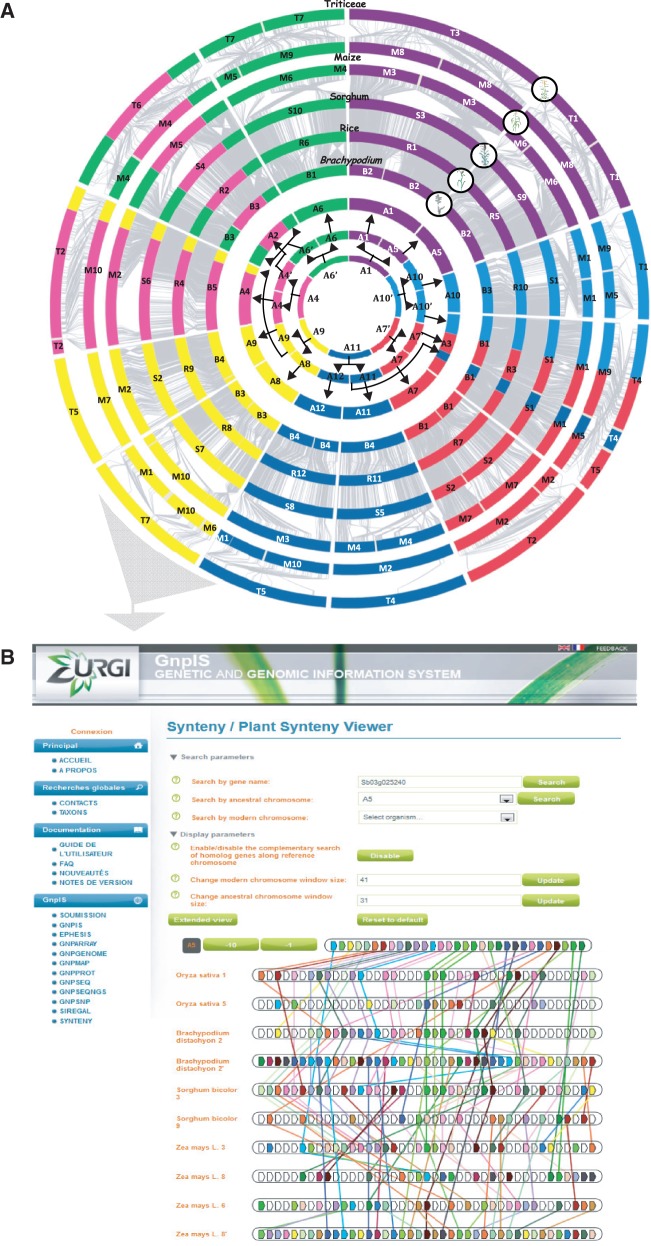
New grass synteny visualization tools. (*A*) Grass synteny circles. The Triticeae, maize, sorghum, *Brachypodium,* and rice chromosomes are represented as concentric circles according to their genome size with the *Brachypodium* as the smallest at the center. The seven chromosome colors refer to the seven ancestral chromosomes (A4 = pink, A5 = purple, A6′ = green, A7′ = red, A8 = yellow, A10 = light blue, A12 = dark blue), and gray lines indicate the orthologous relationships between the modern grass genomes and the seven ancestral chromosomes (inner circles). Black arrows illustrate the ancestral tetraploidization between the ancestral *n* = 7, *n* = 14, and *n* = 12 ancestor intermediates. The Triticeae, maize (double circle), sorghum, and *Brachypodium* chromosome numbers are indicated on the circles. (*B*) PlantSyntenyViewer tool. The entry page of the PlantSyntenyViewer tool in which the setting parameters (search by gene name, ancestral or modern chromosomes) are mentioned at the top and the derived paleogenomics data visualization with AGK (A5 in this screen), rice, maize, sorghum, *Brachypodium* gene conservation (colored connecting lines) at the bottom. The PlantSyntenyViewer tool is available at http://urgi.versailles.inra.fr/synteny-cereal.

The syntenies observed between plant genomes were classically illustrated through a pioneering model of circular consensus genetic maps of grasses, the so-called crop circles, initiated by Mike Gale’s group (Moore et al. 1995), where the genomes were arranged as concentric circles according to their size and syntenic relationships. We recently updated such crop circles using the genome-sequence-based paleogenomics data described previously, suggesting that grasses derived from an *n* = 5 ancestor (Bolot et al. 2009). The crop circles in figure 6*A* clearly illustrate the chromosome-to-chromosome conservation (gray lines between circles as orthologous genes) observed in monocots (involving *Brachypodium*/rice/sorghum/maize/Triticeae), based on the newly characterized *n* = 7 ancestor detailed here. Thus, based on this refined representation of synteny relationships (illustrated with a color code that highlights the ancestral karyotype structure), it is possible to immediately identify the ancestral relationships and origins (WGD, breakage, CFs) of the different chromosomes in each of the five modern grass genomes for any radius of the crop circles (fig. 6*A*). For example, one of the ancestral duplications (between A1 and A5, illustrated in purple) involves orthologous/paralogous modern chromosomes 1–5, 3-6-8, 3-9, and 1-3, respectively, in rice, maize, sorghum, and the Triticeae.

The paleogenomics data presented here, in terms of ancestral genome structures (i.e., protochomosome characterization as well as protogene order inference), associated with a robust comparison of modern genomes, can now be considered as an applied tool to navigate accurately between genomes and transfer genomic informations (i.e., gene structures and functions) from models to grass species of agronomic interest. To do so, we have provided a user-friendly web tool named PlantSyntenyViewer (http://urgi.versailles.inra.fr/synteny-cereal, last accessed December 24, 2013), allowing access to the orthologous, paralogous, and ancestral relationships described in the current article and illustrated in the previous crop circles (fig. 6*B*). Using this tool, it is possible to navigate from one genome to another using a gene name, a modern chromosome nomenclature, or ancestral protochromosome references. This tool offers for the first time in the same screenshot the complete set of identified orthologs and paralogs from the sequenced grass genomes for any considered region or gene of interest. PlantSyntenyViewer thus provides information about the nonredundant ancestral plant gene set that can be used as a platform for the development of conserved orthologous set (COS) markers (Quraishi et al. 2009) to support cross-genome map-based cloning strategies in grasses. Paleogenomics data can greatly simplify and accelerate the identification of useful markers or candidate genes. The relative structural organization of genes is conserved across plant species (the number of exons and introns and positions of individual introns are mostly conserved in the maize, wheat, *Brachypodium*, sorghum, and barley orthologs). This allows the development of intron-spanning PCR-based primers located within conserved exons. A large set of COS markers suitable for plant genome mapping that are highly transferable (as they are derived from a robust synteny relationship between cereals), highly polymorphic (exploiting the greater number of polymorphisms within introns, i.e., SNP), and codominant (as heterozygous haplotypes can be differentiated from homozygous ones) was released for wheat (Pont et al. 2013), showing that comparative genomics-based paleogenomics data available in the web tool PlantSyntenyViewer represents a valuable resource for marker development and trait dissection in grasses (Quraishi, Murat, et al. 2011; Quraishi, Abrouk, et al. 2011; Dibari et al. 2012).

## Conclusions

Precise reconstruction of the ancestral genomes allowed a reconsideration of plant paleohistory, highlighting new evolutionary rules where polyploidy-based chromosomal dominance defines highly plastic sensitive fragments and stable dominant counterparts in any modern genome. The contrasted evolutionary plasticity between these genomic compartments now provides a new working hypothesis, where adaptation (in particular, in response to biotic and abiotic stresses) is possibly partitioned in the modern plant genomes, especially in their sensitive chromosomal compartments.

## Supplementary Material

Supplementary figures S1-S12 are available at *Genome Biology and Evolution* online (http://www.gbe.oxfordjournals.org/).

Supplementary Data
