# Supplementary material for: Shared Subgenome Dominance Following Polyploidization Explains Grass Genome Evolutionary Plasticity from a Seven Protochromosome Ancestor with 16K Protogenes
Source: Genome Biol Evol. 2013 Dec 6;6(1):12–33. doi: 10.1093/gbe/evt200 (PMC3914691; doi:10.1093/gbe/evt200)
Supplement: Supplementary Data [file supp_6_1_12__index.html]

Shared Sub-genome Dominance Following Polyploidization Explains Grass Genome Evolutionary Plasticity from a 7 Protochromosome Ancestor with 16K Protogenes. — Shared Subgenome Dominance Following Polyploidization Explains Grass Genome Evolutionary Plasticity from a Seven Protochromosome Ancestor with 16K Protogenes — Supplementary Data 

# Shared Subgenome Dominance Following Polyploidization Explains Grass Genome Evolutionary Plasticity from a Seven Protochromosome Ancestor with 16K Protogenes

## Supplementary Data

files

**Files in this Data Supplement:**

- Supplementary Data - pdf file
