# Supplementary material for: Shared Subgenome Dominance Following Polyploidization Explains Grass Genome Evolutionary Plasticity from a Seven Protochromosome Ancestor with 16K Protogenes
Source: Genome Biol Evol. 2013 Dec 6;6(1):12–33. doi: 10.1093/gbe/evt200 (PMC3914691; doi:10.1093/gbe/evt200)
Supplement: Supplementary Data [file supp_evt200_2013_revised_GBE_FigS1-S12.pdf]

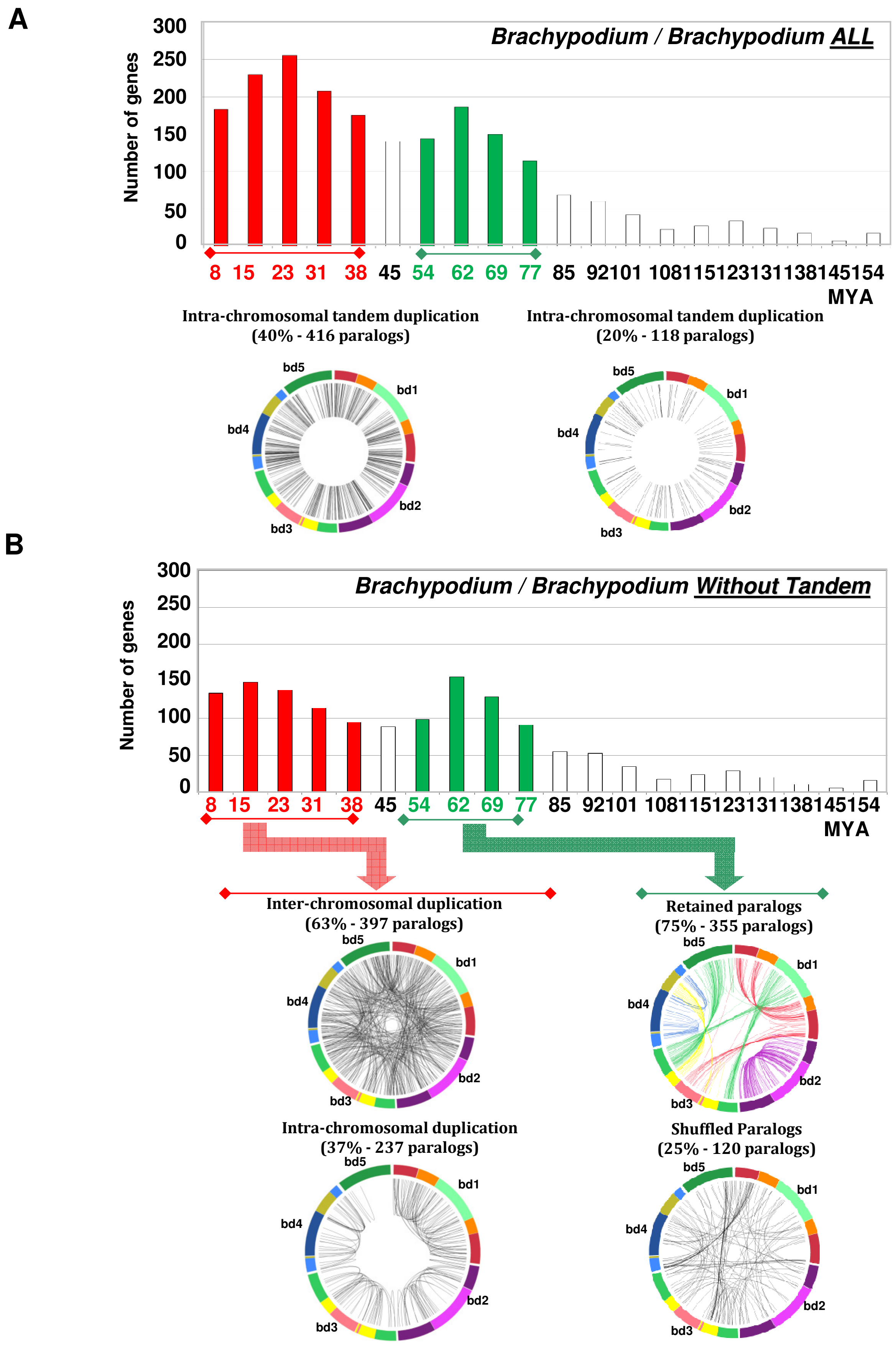
A
Brachypodium / Brachypodium ALL
Number of genes
300
250
200
150
100
50
0
8 15 23 31 38 45 54 62 69 77 85 92101 108115123131138145154
MYA
Intra-chromosomal tandem duplication
(40% - 416 paralogs)
Intra-chromosomal tandem duplication
(20% - 118 paralogs)
bd1
bd2
bd3
bd4
bd5
B
Brachypodium / Brachypodium Without Tandem
Number of genes
300
250
200
150
100
50
0
8 15 23 31 38 45 54 62 69 77 85 92101 108115123131138145154
MYA
Inter-chromosomal duplication
(63% - 397 paralogs)
Retained paralogs
(75% - 355 paralogs)
Intra-chromosomal duplication
(37% - 237 paralogs)
Shuffled Paralogs
(25% - 120 paralogs)

Figure S1

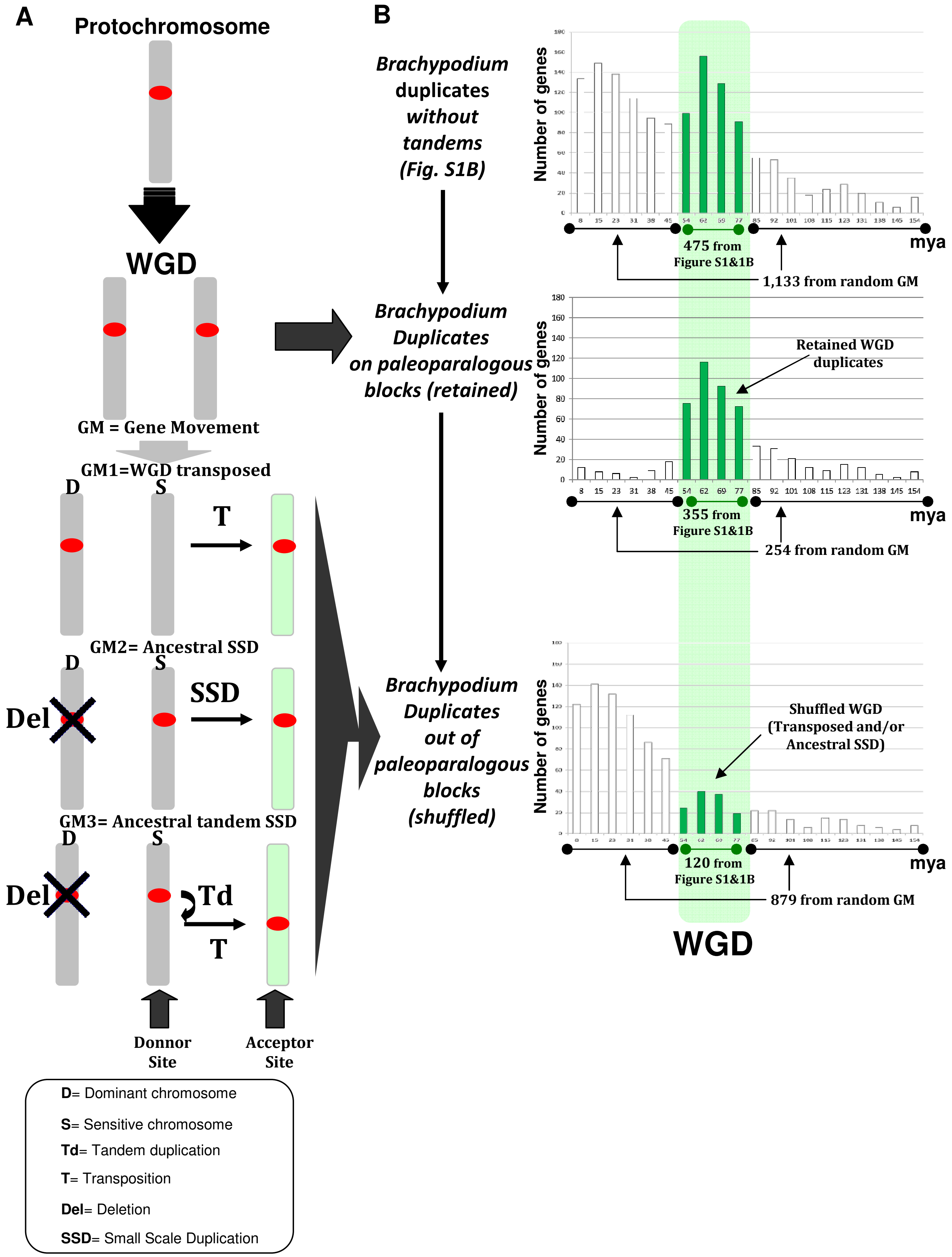
A
Protochromosome
WGD
GM = Gene Movement
GM1=WGD transposed
D
S
T
GM2= Ancestral SSD
D
S
Del
SSD
GM3= Ancestral tandem SSD
D
S
Del
Td
T
Donnor Site
Acceptor Site
D= Dominant chromosome
S= Sensitive chromosome
Td= Tandem duplication
T= Transposition
Del= Deletion
SSD= Small Scale Duplication
B
Brachypodium duplicates without tandems (Fig. S1B)
Number of genes
mya
475 from Figure S1&1B
1,133 from random GM
Brachypodium Duplicates on paleoparalogous blocks (retained)
Number of genes
0
20
40
60
80
100
120
140
160
180
8
15
23
31
38
45
54
62
69
77
85
92
101
108
115
123
131
138
145
154
Retained WGD duplicates
mya
355 from Figure S1&1B
254 from random GM
Brachypodium Duplicates out of paleoparalogous blocks (shuffled)
Number of genes
Shuffled WGD (Transposed and/or Ancestral SSD)
mya
120 from Figure S1&1B
879 from random GM
WGD

Figure S2

Figure S2 (end)

C

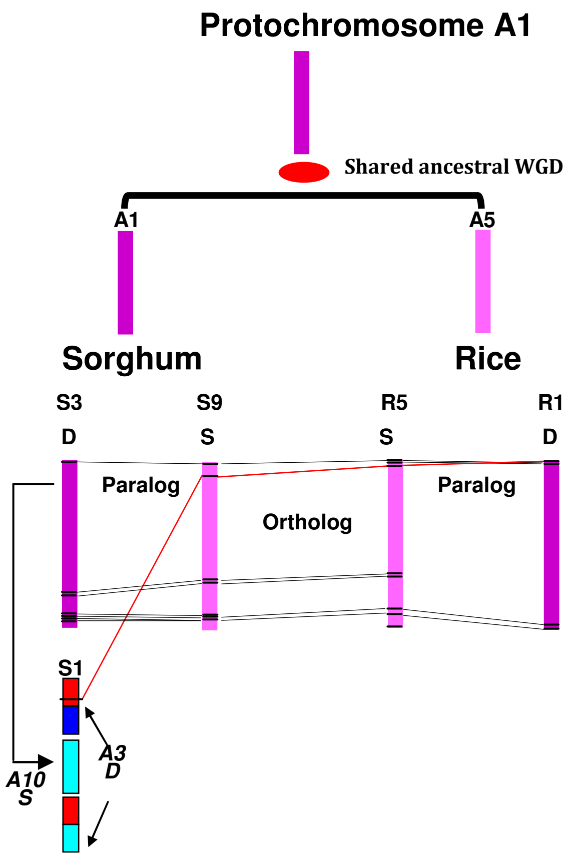

A

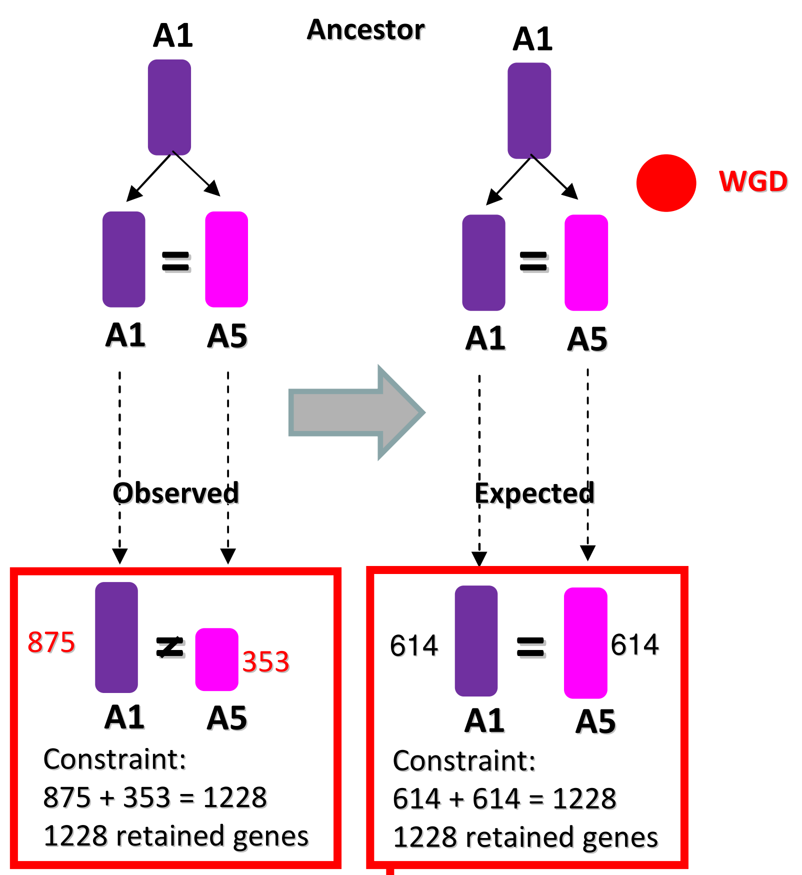

B

		Rice			*Brachypodium*			Sorghum			Maize			TOTAL
		Obs	Exp	χ^2 pvalue	Obs	Exp	χ^2 pvalue	Obs	Exp	χ^2 pvalue	Obs	Exp	χ^2 pvalue	χ^2 pvalue
D	A1	1,107	801,5	2,20E-16	959	685,5	2,20E-16	875	614	2,20E-16	740	524,5	2,20E-16	**8,28E-92**
S	A5	496			412			353			309			
D	A9	497	427	1,66E-06	453	384,5	7,80E-07	396	331	4,36E-07	341	276	3,15E-08	**3,38E-11**
S	A8	357			316			266			211			
D	A11	168	149	0,02772	132	119	0,09192	105	94	0,1086	76	66,5	0,09945	**0,0901621**
S	A12	130			106			83			57			
D	A4	571	673,5	2,33E-08	507	588,5	2,02E-06	447	545	2,91E-09	163	356	2,20E-16	**4,04E-32**
D+S	A2	776			670			643			549			
S	A6	366	571	2,20E-16	325	497,5	2,20E-16	286	464,5	2,20E-16	260	404,5	2,20E-16	**1,08E-54**
S	A7	441	655,5	2,20E-16	375	566	2,20E-16	272	472	2,20E-16	301	456,5	2,20E-16	**9,53E-59**
D+D	A3	870			757			672			612			
S	A10	323	596,5	2,20E-16	258	507,5	2,20E-16	214	443	2,20E-16	175	393,5	2,20E-16	**2,14E-105**

Figure S3

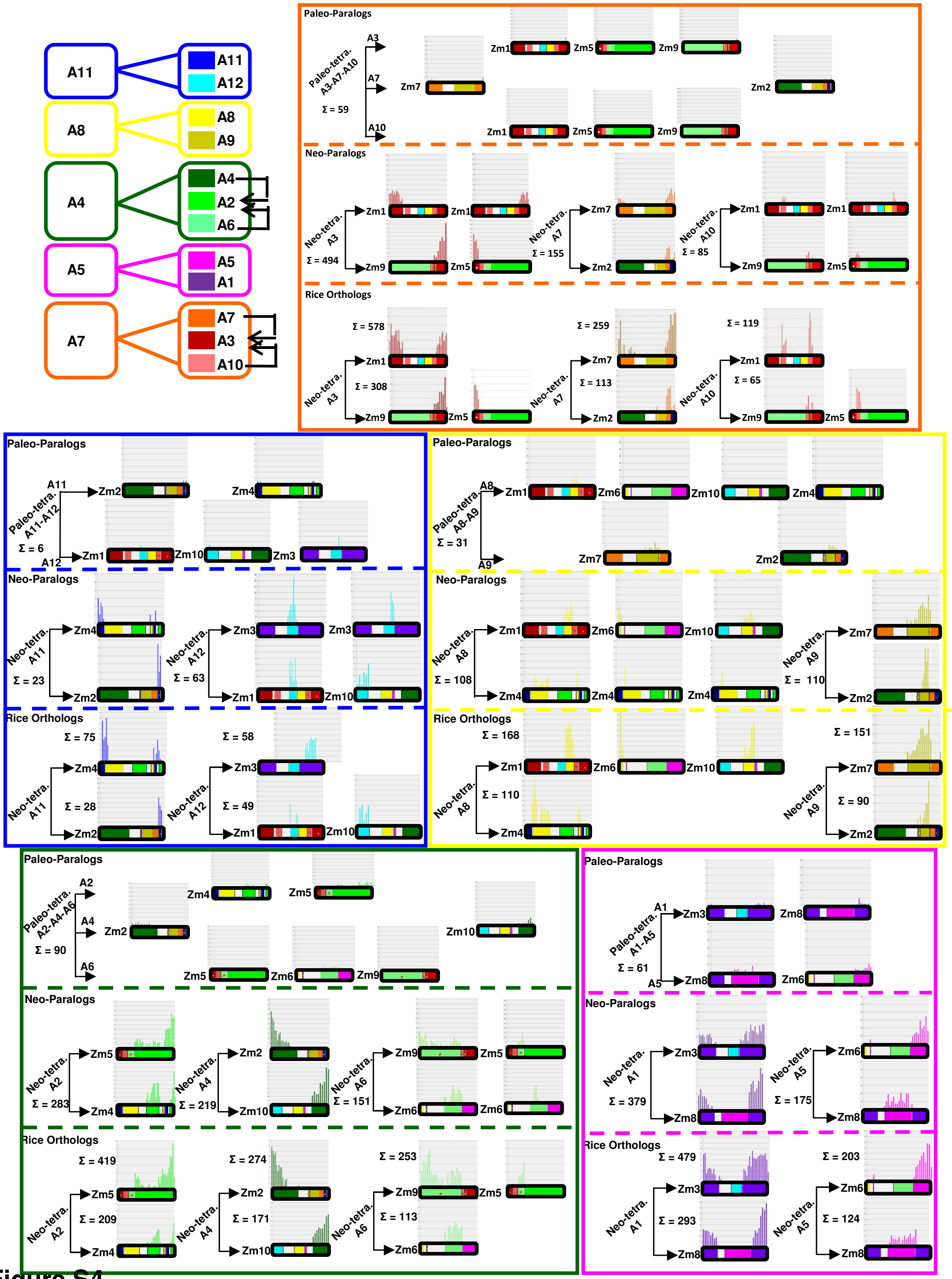

A11
A12
A8
A9
A4
A2
A6
A5
A1
A7
A3
A10
Paleo-Paralogs
Neo-Paralogs
Rice Orthologs
Paleo-tetra. A3-A7-A10
Σ = 59
Neo-tetra. A3
Σ = 494
Neo-tetra. A7
Σ = 155
Neo-tetra. A10
Σ = 85
Σ = 578
Σ = 308
Σ = 259
Σ = 113
Σ = 119
Σ = 65
Paleo-tetra. A11-A12
Σ = 6
Neo-tetra. A11
Σ = 23
Neo-tetra. A12
Σ = 63
Σ = 75
Σ = 28
Σ = 58
Σ = 49
Paleo-tetra. A8-A9
Σ = 31
Neo-tetra. A8
Σ = 108
Neo-tetra. A9
Σ = 110
Σ = 168
Σ = 110
Σ = 151
Σ = 90
Paleo-tetra. A2-A4-A6
Σ = 90
Neo-tetra. A2
Σ = 283
Neo-tetra. A4
Σ = 219
Neo-tetra. A6
Σ = 151
Σ = 419
Σ = 209
Σ = 274
Σ = 171
Σ = 253
Σ = 113
Paleo-tetra. A1-A5
Σ = 61
Neo-tetra. A1
Σ = 379
Neo-tetra. A5
Σ = 175
Σ = 479
Σ = 293
Σ = 203
Σ = 124
Zm1
Zm2
Zm3
Zm4
Zm5
Zm6
Zm7
Zm8
Zm9
Zm10

Figure S4

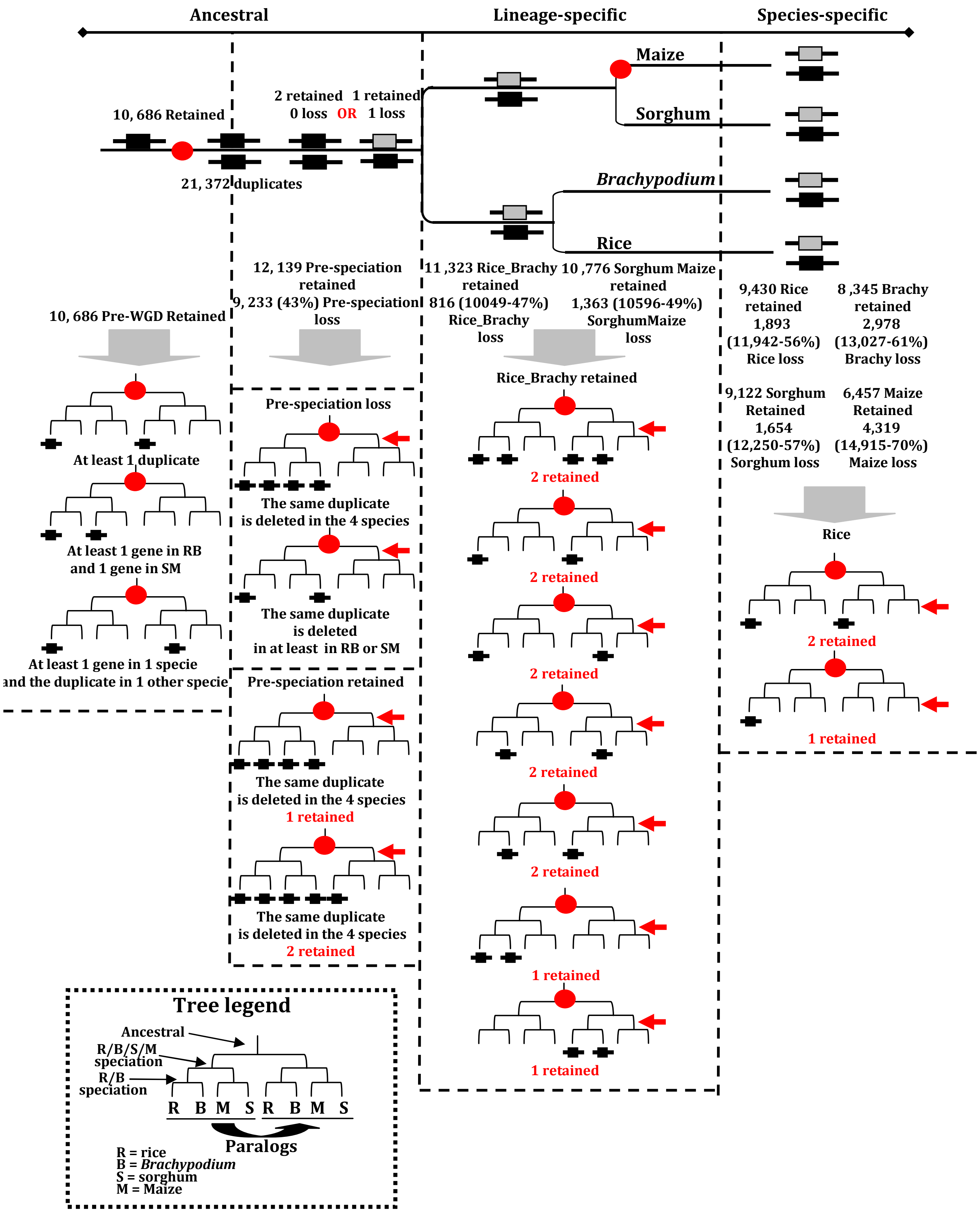
Ancestral
Lineage-specific
Species-specific
10, 686 Retained
2 retained 0 loss
OR
1 retained 1 loss
21, 372 duplicates
Maize
Sorghum
Brachypodium
Rice
12, 139 Pre-speciation retained
9, 233 (43%) Pre-speciation loss
11 ,323 Rice_Brachy retained
816 (10049-47%) Rice_Brachy loss
10 ,776 Sorghum Maize retained
1,363 (10596-49%) SorghumMaize loss
9,430 Rice retained
1,893 (11,942-56%) Rice loss
8 ,345 Brachy retained
2,978 (13,027-61%) Brachy loss
9,122 Sorghum Retained
1,654 (12,250-57%) Sorghum loss
6,457 Maize Retained
4,319 (14,915-70%) Maize loss
10, 686 Pre-WGD Retained
At least 1 duplicate
At least 1 gene in RB and 1 gene in SM
At least 1 gene in 1 specie and the duplicate in 1 other specie
Pre-speciation loss
The same duplicate is deleted in the 4 species
The same duplicate is deleted in at least in RB or SM
Pre-speciation retained
The same duplicate is deleted in the 4 species
1 retained
The same duplicate is deleted in the 4 species
2 retained
Rice_Brachy retained
2 retained
2 retained
2 retained
2 retained
2 retained
1 retained
1 retained
Rice
2 retained
1 retained
Tree legend
Ancestral
R/B/S/M speciation
R/B speciation
R B M S R B M S
Paralogs
R = rice
B = Brachypodium
S = sorghum
M = Maize

Figure S5

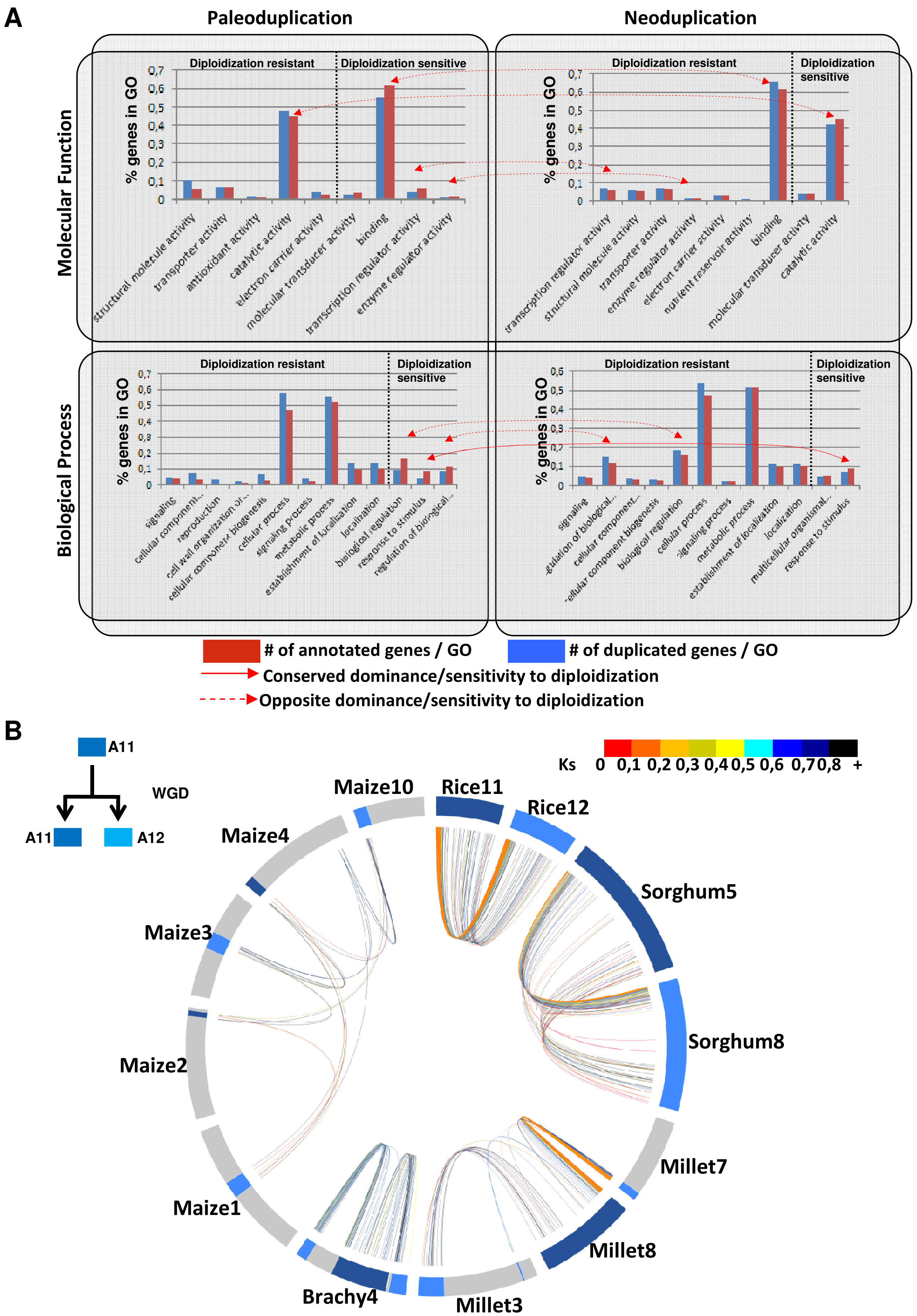
A
Paleoduplication
Neoduplication
Molecular Function
Biological Process
Diploidization resistant
Diploidization sensitive
% genes in GO
structural molecule activity
transporter activity
antioxidant activity
catalytic activity
electron carrier activity
molecular transducer activity
binding
transcription regulator activity
enzyme regulator activity
nutrient reservoir activity
signaling
cellular component...
reproduction
cell wall organization or...
cellular component biogenesis
cellular process
signaling process
metabolic process
establishment of localization
localization
biological regulation
response to stimulus
regulation of biological...
multicellular organismal...
# of annotated genes / GO
# of duplicated genes / GO
Conserved dominance/sensitivity to diploidization
Opposite dominance/sensitivity to diploidization
B
A11
WGD
A11
A12
Ks 0 0,1 0,2 0,3 0,4 0,5 0,6 0,7 0,8 +
Maize10
Rice11
Rice12
Sorghum5
Sorghum8
Millet7
Millet8
Millet3
Brachy4
Maize1
Maize2
Maize3
Maize4

Figure S6

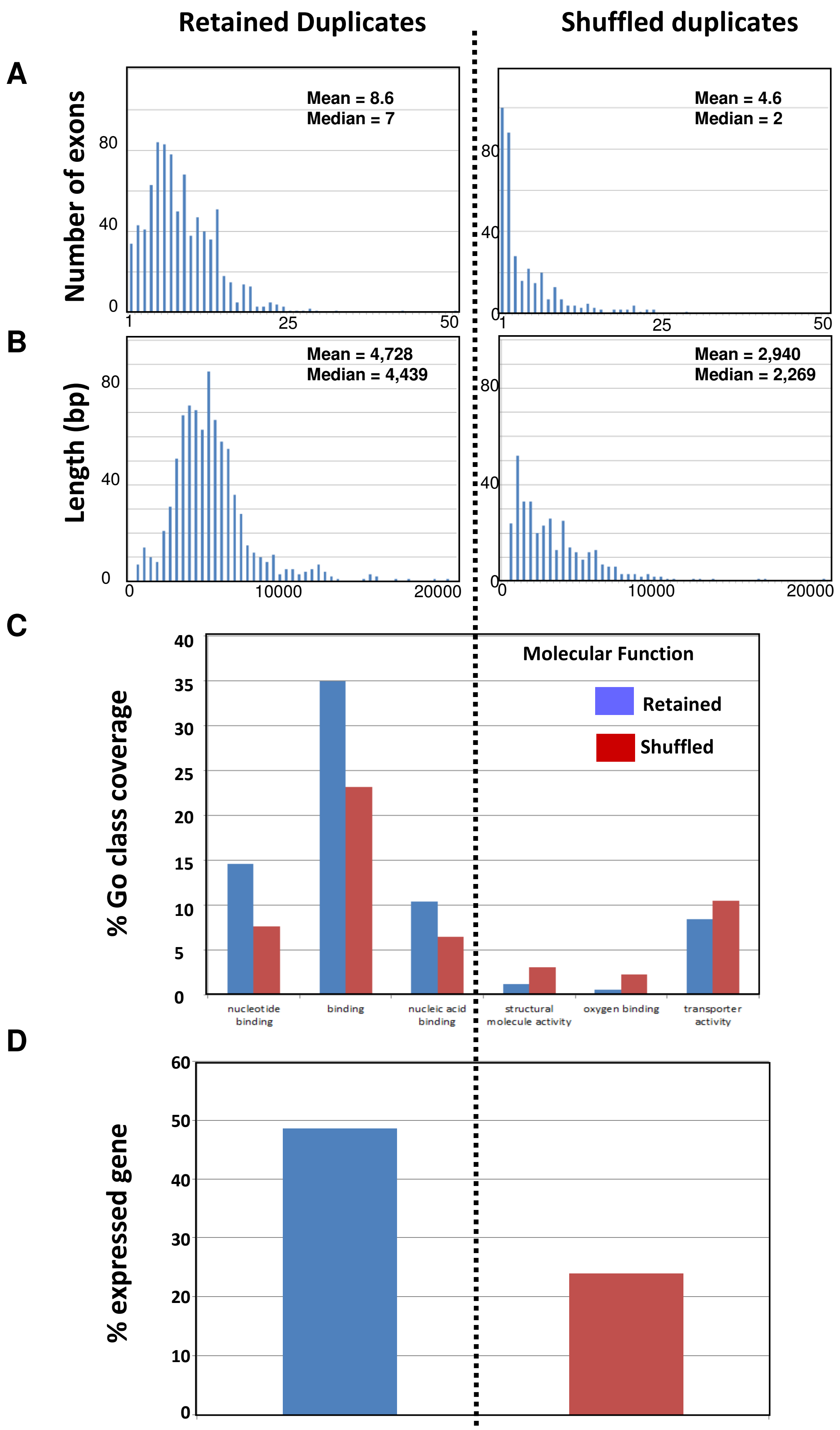
Retained Duplicates
Shuffled duplicates
A
Number of exons
Mean = 8.6
Median = 7
Mean = 4.6
Median = 2
B
Length (bp)
Mean = 4,728
Median = 4,439
Mean = 2,940
Median = 2,269
C
% Go class coverage
Molecular Function
Retained
Shuffled
nucleotide binding
binding
nucleic acid binding
structural molecule activity
oxygen binding
transporter activity
D
% expressed gene

Figure S7

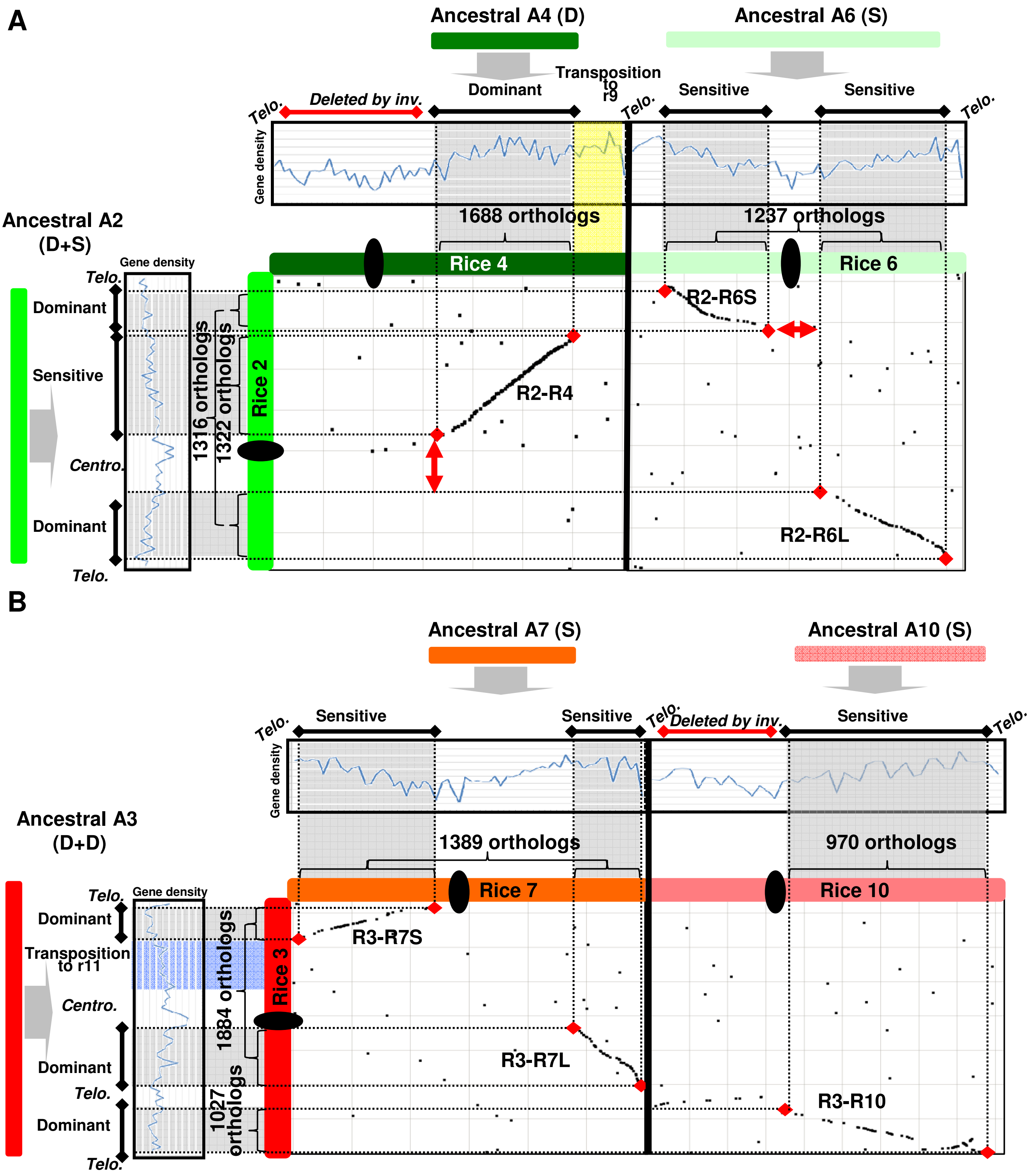
A
Ancestral A4 (D)
Ancestral A6 (S)
Transposition to r9
Dominant
Sensitive
Sensitive
Deleted by inv.
Telo.
Telo.
Telo.
Gene density
1688 orthologs
1237 orthologs
Ancestral A2 (D+S)
Gene density
Rice 4
Rice 6
Telo.
Dominant
Sensitive
Centro.
Dominant
Telo.
1316 orthologs
1322 orthologs
Rice 2
R2-R6S
R2-R4
R2-R6L
B
Ancestral A7 (S)
Ancestral A10 (S)
Sensitive
Sensitive
Telo.
Deleted by inv.
Sensitive
Telo.
Telo.
Gene density
1389 orthologs
970 orthologs
Ancestral A3 (D+D)
Rice 7
Rice 10
Telo.
Gene density
Dominant
Transposition to r11
Centro.
Dominant
Telo.
Dominant
Telo.
1884 orthologs
1027 orthologs
Rice 3
R3-R7S
R3-R7L
R3-R10

Figure S8

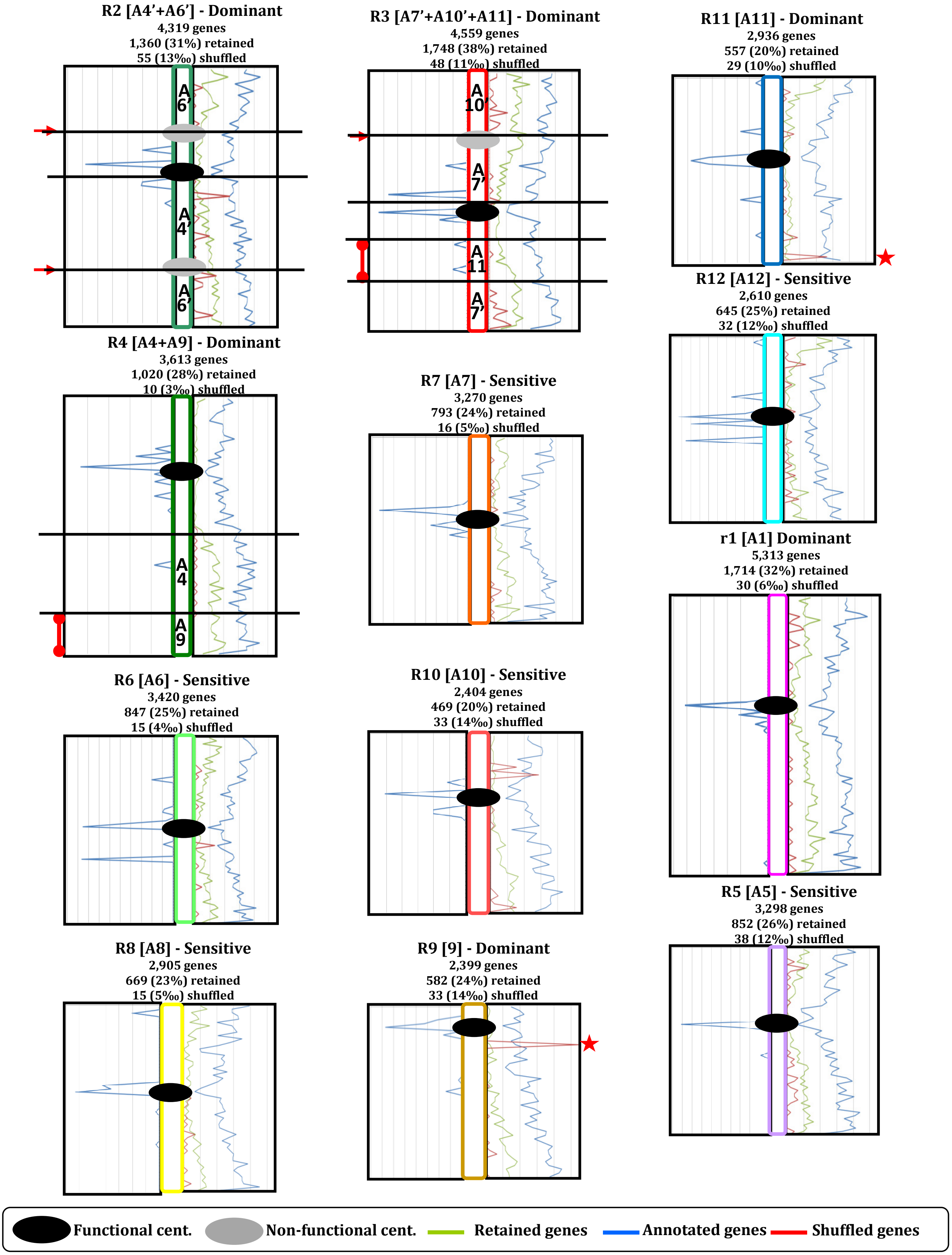
R2 [A4'+A6'] - Dominant
4,319 genes
1,360 (31%) retained
55 (13‰) shuffled
A 6'
A 4'
A 6'
R3 [A7'+A10'+A11] - Dominant
4,559 genes
1,748 (38%) retained
48 (11‰) shuffled
A 10'
A 7'
A 11
A 7'
R11 [A11] - Dominant
2,936 genes
557 (20%) retained
29 (10‰) shuffled
R4 [A4+A9] - Dominant
3,613 genes
1,020 (28%) retained
10 (3‰) shuffled
A 4
A 9
R7 [A7] - Sensitive
3,270 genes
793 (24%) retained
16 (5‰) shuffled
R12 [A12] - Sensitive
2,610 genes
645 (25%) retained
32 (12‰) shuffled
r1 [A1] Dominant
5,313 genes
1,714 (32%) retained
30 (6‰) shuffled
R6 [A6] - Sensitive
3,420 genes
847 (25%) retained
15 (4‰) shuffled
R10 [A10] - Sensitive
2,404 genes
469 (20%) retained
33 (14‰) shuffled
R5 [A5] - Sensitive
3,298 genes
852 (26%) retained
38 (12‰) shuffled
R8 [A8] - Sensitive
2,905 genes
669 (23%) retained
15 (5‰) shuffled
R9 [9] - Dominant
2,399 genes
582 (24%) retained
33 (14‰) shuffled
Functional cent.
Non-functional cent.
Retained genes
Annotated genes
Shuffled genes

Figure S9

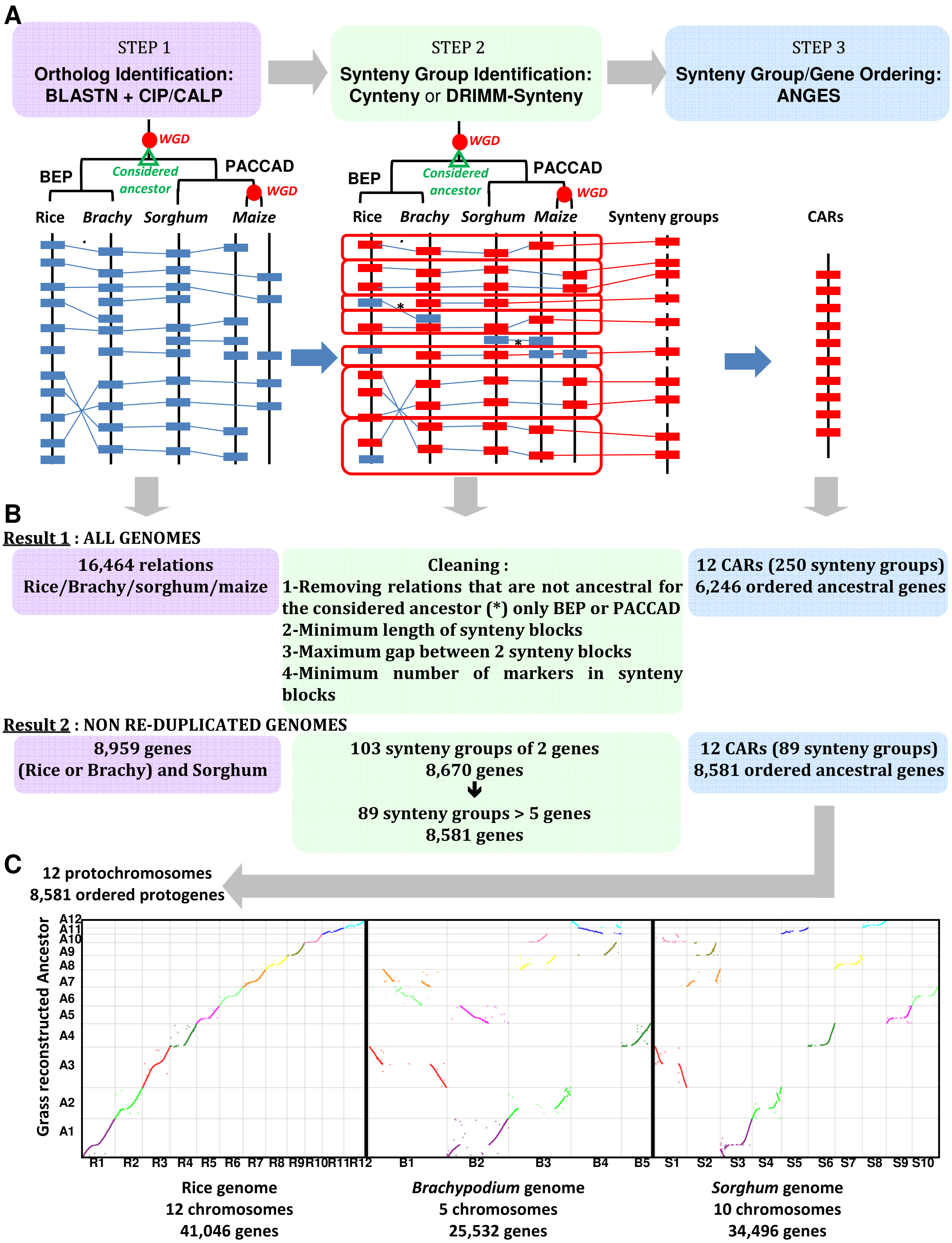

A
STEP 1
Ortholog Identification:
BLASTN + CIP/CALP
STEP 2
Synteny Group Identification:
Cynteny or DRIMM-Synteny
STEP 3
Synteny Group/Gene Ordering:
ANGES
WGD
Considered ancestor
BEP
PACCAD
WGD
Rice
Brachy
Sorghum
Maize
Synteny groups
CARs
B
Result 1 : ALL GENOMES
16,464 relations
Rice/Brachy/sorghum/maize
Cleaning :
1-Removing relations that are not ancestral for the considered ancestor (*) only BEP or PACCAD
2-Minimum length of synteny blocks
3-Maximum gap between 2 synteny blocks
4-Minimum number of markers in synteny blocks
12 CARs (250 synteny groups)
6,246 ordered ancestral genes
Result 2 : NON RE-DUPLICATED GENOMES
8,959 genes
(Rice or Brachy) and Sorghum
103 synteny groups of 2 genes
8,670 genes
89 synteny groups > 5 genes
8,581 genes
12 CARs (89 synteny groups)
8,581 ordered ancestral genes
C
12 protochromosomes
8,581 ordered protogenes
Grass reconstructed Ancestor
A12
A11
A10
A9
A8
A7
A6
A5
A4
A3
A2
A1
R1 R2 R3 R4 R5 R6 R7 R8 R9 R10 R11 R12
B1 B2 B3 B4 B5
S1 S2 S3 S4 S5 S6 S7 S8 S9 S10
Rice genome
12 chromosomes
41,046 genes
Brachypodium genome
5 chromosomes
25,532 genes
Sorghum genome
10 chromosomes
34,496 genes

Figure S10

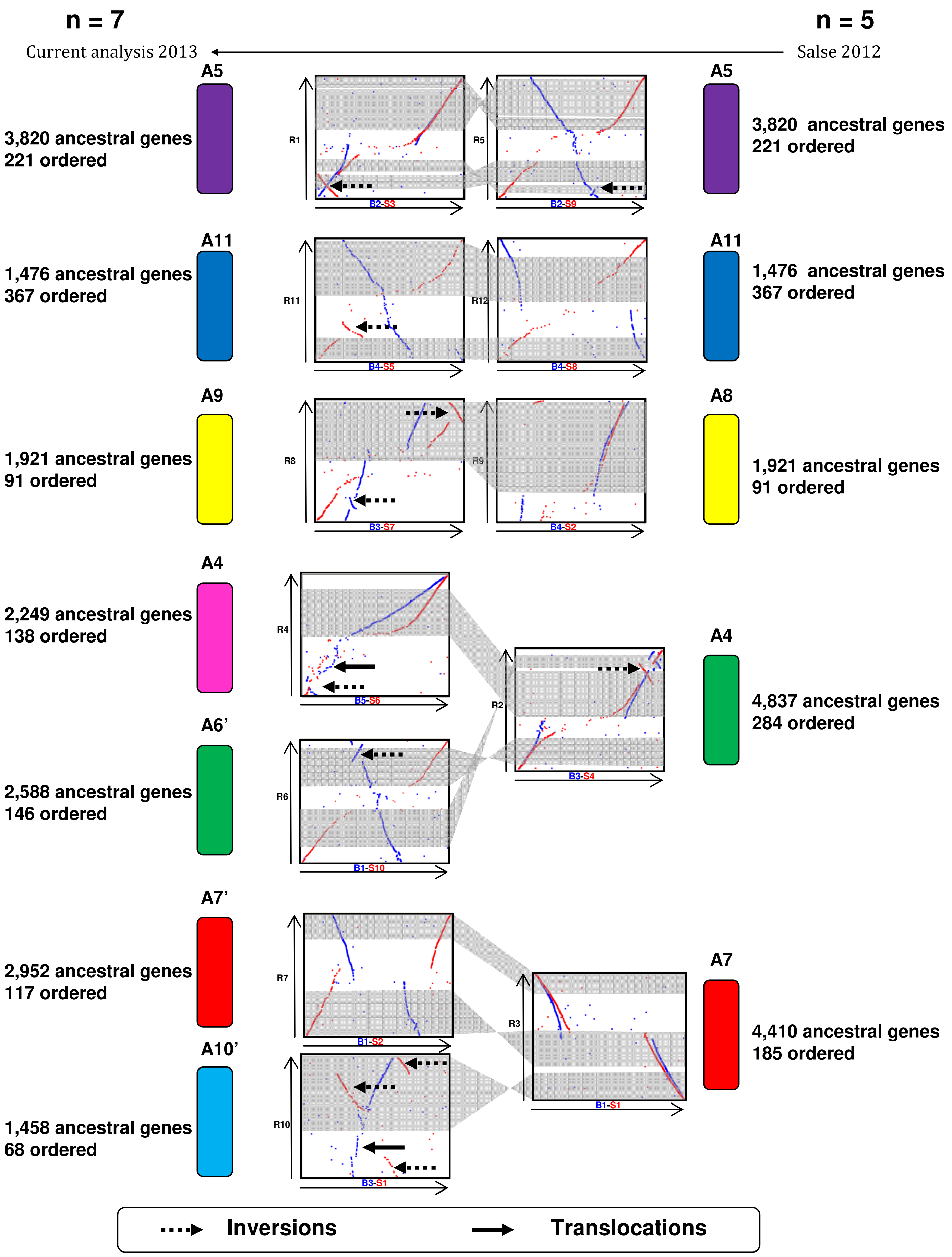

n = 7
Current analysis 2013
n = 5
Salse 2012
A5
3,820 ancestral genes
221 ordered
R1
B2-S3
R5
B2-S9
A5
3,820 ancestral genes
221 ordered
A11
1,476 ancestral genes
367 ordered
R11
B4-S5
R12
B4-S8
A11
1,476 ancestral genes
367 ordered
A9
1,921 ancestral genes
91 ordered
R8
B3-S7
R9
B4-S2
A8
1,921 ancestral genes
91 ordered
A4
2,249 ancestral genes
138 ordered
R4
B5-S6
A6'
2,588 ancestral genes
146 ordered
R6
B1-S10
R2
B3-S4
A4
4,837 ancestral genes
284 ordered
A7'
2,952 ancestral genes
117 ordered
R7
B1-S2
A10'
1,458 ancestral genes
68 ordered
R10
B3-S1
R3
B1-S1
A7
4,410 ancestral genes
185 ordered
Inversions
Translocations

Figure S11

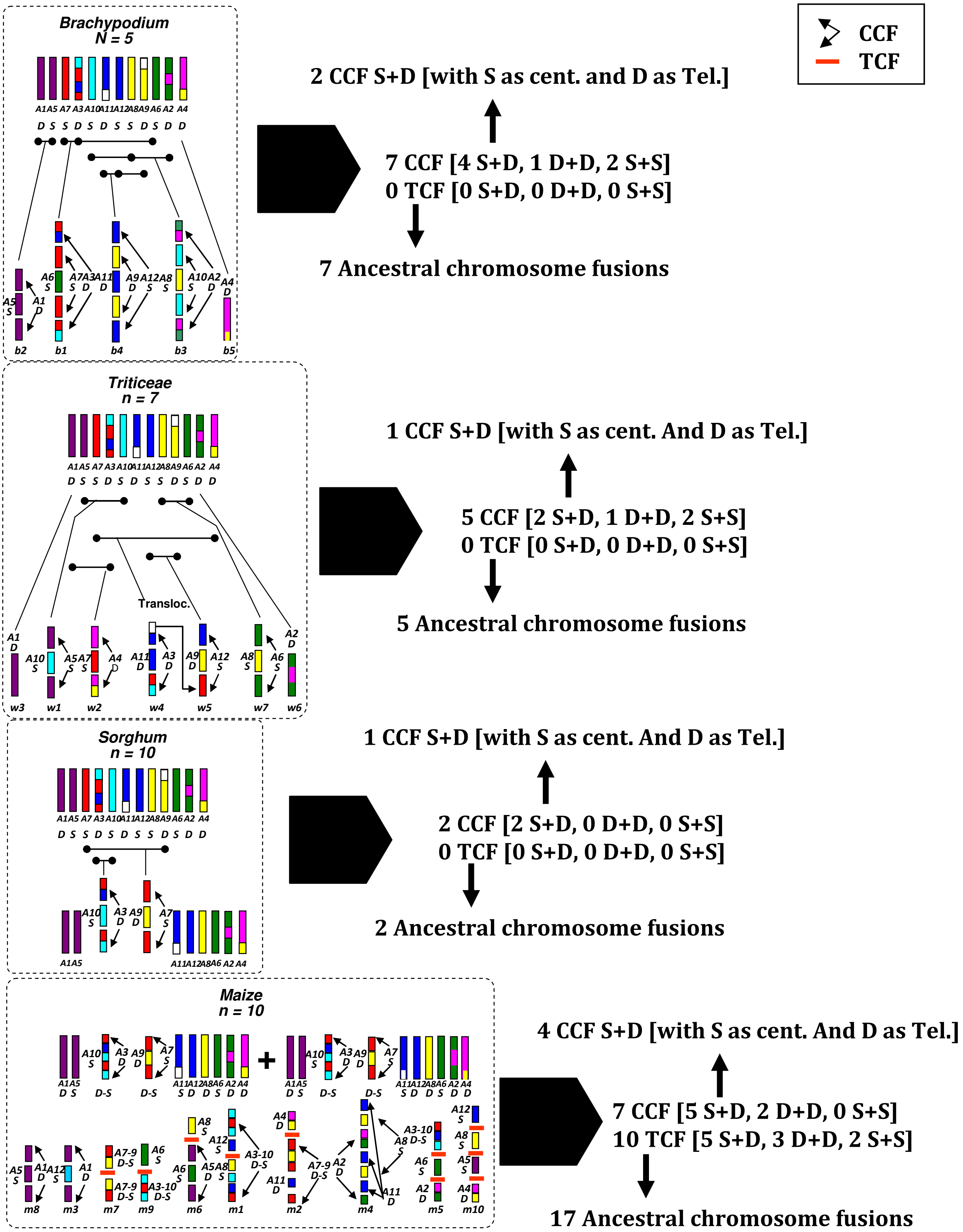
CCF
TCF
Brachypodium
N = 5
2 CCF S+D [with S as cent. and D as Tel.]
7 CCF [4 S+D, 1 D+D, 2 S+S]
0 TCF [0 S+D, 0 D+D, 0 S+S]
7 Ancestral chromosome fusions
Triticeae
n = 7
Transloc.
1 CCF S+D [with S as cent. And D as Tel.]
5 CCF [2 S+D, 1 D+D, 2 S+S]
0 TCF [0 S+D, 0 D+D, 0 S+S]
5 Ancestral chromosome fusions
Sorghum
n = 10
1 CCF S+D [with S as cent. And D as Tel.]
2 CCF [2 S+D, 0 D+D, 0 S+S]
0 TCF [0 S+D, 0 D+D, 0 S+S]
2 Ancestral chromosome fusions
Maize
n = 10
4 CCF S+D [with S as cent. And D as Tel.]
7 CCF [5 S+D, 2 D+D, 0 S+S]
10 TCF [5 S+D, 3 D+D, 2 S+S]
17 Ancestral chromosome fusions

Figure S12
